# Three-dimensional reconstruction and NURBS-based structured meshing of coronary arteries from the conventional X-ray angiography projection images

**DOI:** 10.1038/s41598-018-19440-9

**Published:** 2018-01-26

**Authors:** Arso M. Vukicevic, Serkan Çimen, Nikola Jagic, Gordana Jovicic, Alejandro F. Frangi, Nenad Filipovic

**Affiliations:** 1 Faculty of Engineering Sciences, University of Kragujevac, Kragujevac, Serbia; 2 Center for Computational Imaging & Simulation Technologies in Biomedicine, Electronic & Electrical Engineering Department, The University of Sheffield, Sheffield, UK; 3 Faculty of Medicine, University of Kragujevac, Kragujevac, Serbia; 4 Research and Development Center for Bioengineering, Kragujevac Kragujevac, Serbia; 5 Faculty of Information Technology, Belgrade Metropolitan University, Belgrade, Serbia

## Abstract

Despite its two-dimensional nature, X-ray angiography (XRA) has served as the gold standard imaging technique in the interventional cardiology for over five decades. Accordingly, demands for tools that could increase efficiency of the XRA procedure for the quantitative analysis of coronary arteries (CA) are constantly increasing. The aim of this study was to propose a novel procedure for three-dimensional modeling of CA from uncalibrated XRA projections. A comprehensive mathematical model of the image formation was developed and used with a robust genetic algorithm optimizer to determine the calibration parameters across XRA views. The frames correspondences between XRA acquisitions were found using a partial-matching approach. Using the same matching method, an efficient procedure for vessel centerline reconstruction was developed. Finally, the problem of meshing complex CA trees was simplified to independent reconstruction and meshing of connected branches using the proposed nonuniform rational B-spline (NURBS)-based method. Because it enables structured quadrilateral and hexahedral meshing, our method is suitable for the subsequent computational modelling of CA physiology (i.e. coronary blood flow, fractional flow reverse, virtual stenting and plaque progression). Extensive validations using digital, physical, and clinical datasets showed competitive performances and potential for further application on a wider scale.

## Introduction

Coronary arteries (CA) are small and dynamic vessels that branch from the aorta and supply myocardium with oxygen-rich blood. According to the clinical reports, coronary artery disease (CAD) represents leading cause of death in the developed world^1^. Briefly, CAD features CA wall stiffening and lumen narrowing, which may be diagnosed using various imaging modalities. Despite its invasiveness and two-dimensional nature, X-ray angiography (XRA) has served as the gold standard technique in interventional cardiology for over five decades. Moreover, XRA has facilitated many of the catheter-based cardiovascular procedures developed in the meanwhile (fractional flow reserve, angioplasty, intravascular ultrasound, optical coherence tomography and stenting). Consequently, there are increasing needs for tools that could enable accurate 3D quantification of CAD using the standard monoplane (when at least two views are available) and biplane (two views are acquired simultaneously) XRA devices^2,3^.

**Figure 1:**
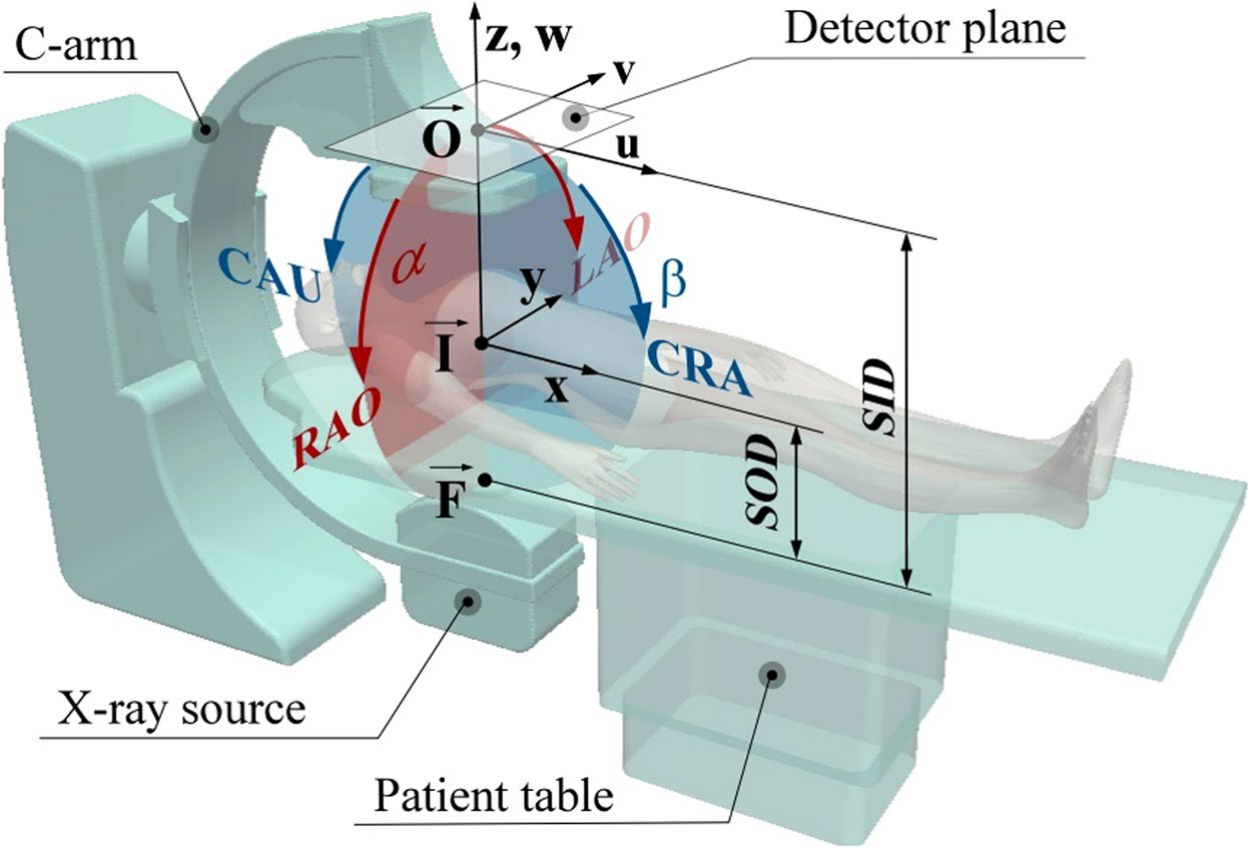
Construction of a conventional monoplane X-ray angiography device.

Typical XRA device consists of the X-ray source ($\overrightarrow{{\bf{F}}}$) and the image detector ($\overrightarrow{{\bf{O}}}$) mounted on the mobile C-arm that can be rotated around an object, which is placed on the mobile patient table and subjected for the imaging. The sketch on Fig. 1 shows the C-arm in its neutral (vertical), *anterior-posterior* (AP) position, when the X-ray source is below the patient table. The XRA imaging is performed by a trained physician, who uses a catheter to inject the radio-opaque contrast agent into the arteries and positions the device to obtain optimal two-dimension projections of the vasculature. Particularly, one could rotate C-arm around a point called isocenter ($\overrightarrow{{\bf{I}}}$) by using two rotations: positioner primary (*α*) and secondary (*β*) angle. The angle *α* may vary from +180° at the patient left hand side (LAO) to −180° at the patient right hand side (RAO). The secondary angle *β* range from +90° (cranial – CRA) to −90° (caudial – CAU). And one could change the distances between the patient and the X-ray source (SOD) as well between the X-ray source and the detector-plane (SID). Together with the images, these parameters are stored within the DICOM file header and represent routinely acquired data during the XRA imaging.

### Related work

The majority of the available methods for reconstructing CA from XRA are semi-automatic and consist of these five steps: (1) pairing of frames acquired from different views; (2) vessel segmentation, decomposition and tracking in the XRA dynamic runs; (3) calibration of the parameters defining the device orientations; (4) modeling of CA centerline from its synchronized segmentations; and (5) reconstruction of the CA tree surface. In this part of the section, we briefly review state of the art approaches for solving these reconstruction tasks (see also Table 1).

**Table 1:** Comparative overview of the features provided by studies.

Method	Calibration	Centerline	Lumen approximation cross-section (# views)	4D	Delivered mesh format	Surface validation approach (referent)
Proposed	Opt. I & E	PM (B-Spline)	C (2), E (2–4), P (4+)	+	PS (NURBS, TRI, TET, HEX, QUAD)	DP QA (GT), PP QA (CT) RP QA (CT)
Chen & Carroll^7^	Opt. E	EM (poly)	C (2)	—	PCT	RP VA
Cañero *et al*.^10^	+	AC (poly)	—	—	N/A	—
Chen & Carroll^16^	Opt. E	EM (poly)	C (2)	+	PCT	RP VA
Andriotis *et al*.^18^	Opt. E	EM (poly)	C (2)	+	PCT	PP QA (CT), RP QA (CT)
Yang *et al*.^9^	Opt. I & E	EM (poly)	C (2)	—	PCT	PP VA (GT), RP VA
Zheng *et al*.^14^	Opt. E	AC (poly)	—	+	N/A	—
Yang *et al*.^22^	Opt. I & E	AC (poly)	N/A	+	PCT	DP QA (GT), RP VA
Cong *et al*.^12^	+	AC (poly)	E (2–5)	—	PCT	DP QA (GT), RP VA

I-intrinsic, E-extrinsic, Opt.-optimization, + -precalibrated, AC-active contours, PM-partial matching, EM-epipolar matching, C-circle, E-elipse, P-polyline, PS-parametric surface, PCT-point cloud triangulation, DP-digital phantom, PP-physical phantom, RP-real patient, GT-ground truth, CT-computed tomography, VA-visual assessment, QA-quantitative assessment.

### XRA device calibration

The first attempts to reconstruct CAs from their XRA projections assumed that only the extrinsic device parameters from the DICOM tags are required^4–6^. Since the angles and distances stored in DICOM headers are of limited accuracy, various methods based on optimization of both gantry extrinsic and intrinsic parameters have been proposed to avoid dependence on nonstandard calibration equipment. Chen and Carroll aimed at the minimization of errors caused by the movement of isocenter between two projections^7^. Besides the calibration of pincushion distortion, Shechter *et al*. investigated the influence of in-plane affine motions during vessel projection on the image intensifier^8^. Yang *et al*. ignored both pincushion distortion (which is appropriate for the flat panel detectors) and in-plane rotation of image detector, but they included movement of the patient table, intensifier in-plane translation and skewness (in x direction) within the projection matrix^9^.

### Centerlines and surface reconstruction

Regarding the 3D reconstruction of vessel centerlines, various deformable models^10–12^ and dynamic programming methods^8,13,14^ are suggested to avoid the epipolar geometry problem when a curve’s point from one view intersects the corresponding centerline on other views more than once^15^. Obtaining a 3D vessel surface from its 2D projections remains a nontrivial task. Although pioneering studies on the topic used circles and ellipses to fit the cross-sections^16,17^, a recent study of Yang *et al*.^9^ proposed a model for fitting elliptical patches. Recently, some studies have suggested various weighting techniques for obtaining a complex polygonal approximation of the lumen in situations when the XRA projection of vessel 3D cross-sections at an arbitrary centerline point does not match 2D cross-section orientation at the corresponding point in the intensifier plane^18–20^.

### XRA frames gating and temporal reconstruction of CAs

Reconstructing a sequence of XRA frames requires finding the correspondence between the frames acquired from different viewpoints, which is commonly addressed by using ECG-gating or by using a bi-plane XRA device. Beside using additional in-plane compensation of complex cardiac motions^21^, recent studies have assumed that a referent static 3D reconstruction is performed from the corresponding frames and then CA is tracked over time^13,14^. The second approach independently reconstructed CA sequences and then used various algorithms for temporal matching of the CA tree – for example, starting from the time-point with the least global motion^19^.

The summarized overview of the previous studies that are comparable with ours in terms of input requirements and aims are given in Table 1. As it may be noted, the majority of previous contributions in each area were tackled independently and separately, and so far, only few studies integrated all the five steps^16,18,22^. Moreover, we found that previous studies overlook the recent breakthrough of numerical simulations into the field of biomedicine^23^.

The bottleneck for further advancements and wider scale applications of biomedical simulations of CA physiology represents improving their compatibility with the imaging procedures, which commonly deliver reconstruction results as a triangulated point cloud mesh. Since the point-cloud meshes are of fixed density and unguaranteed quality, they commonly require an afterwards manual editing (surpassing the time and effort invested into performing the reconstruction or the subsequent numerical simulation). Therefore, the aim of the present study was to propose a novel approach that will integrate all five reconstruction steps and ease using the reconstruction results for the further *in-silico* studies of CA physiology. Considering these goals, the main contributions of the present study are: (1) the robust mathematical model of image formation integrates previous contributions reported separately in recent literature; (2) parameters that specify the orientation of the gantry were identified using robust genetic algorithm; (3) temporal gating of XRA frames acquired from different views was performed using an elastic partial-matching method; (4) the same method was applied for CA centerlines reconstruction, resulting in efficient point-to-point correspondence between the views; (5) the vascular lumen is reconstructed using a NURBS-based method that simplifies further numerical modeling of CA physiology. Finally, the methods are suitable for the parallelization since the CA tree meshing is reduced to independent meshing of connected branches.

## Methods

### Inputs and data structures

To differentiate type of data used over the manuscript, we adopted this nomenclature (see S1 Appendix):

**Table:** 

**x**	–	Data vector-array,
**X**	–	Matrix,
$\overrightarrow{{\bf{x}}}$	–	Point in 2D space (at X-ray plane detector),
$\overrightarrow{{\bf{X}}}$	–	Point in 3D space,
x	–	Constant,
*x*	–	Scalar variable,
X()	–	Scalar function, result is scalar variable (*x*),
$\overrightarrow{{\rm{x}}}()$	–	Vector function, result is point in 2D ($\overrightarrow{{\bf{x}}}$),
$\overrightarrow{{\rm{X}}}()$	–	Vector function, result is point in 3D space ($\overrightarrow{{\bf{X}}}$).

where the temporal indices are assumed to be superscript and the spatial indices are given as subscripts (i.e. ${{\rm{X}}}_{{\rm{sequence}}\,\mathrm{number},\,{\rm{sample}}\,{\rm{ID}}\,{\rm{from}}\,{\rm{current}}\,{\rm{frame}}}^{{\rm{frame}}\,{\rm{number}}}$).

**Figure 2:**
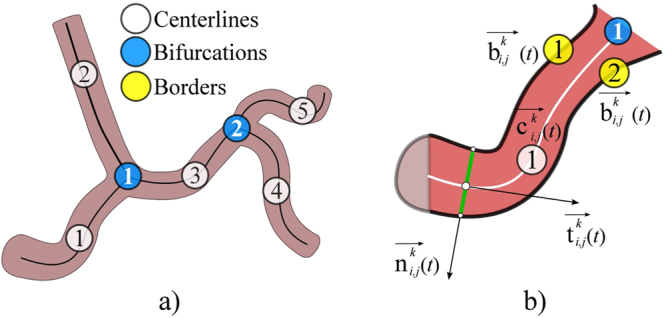
Sketch of data extracted from XRA image. (**a**) CA tree decomposition to connected branches. (**b**) Data extracted from a single CA branch.

Our procedure requires two sequences of XRA images acquired from different viewpoints (the pseudo-code is given in S2 Appendix). After picking the pair of end-diastole frames, a couple (*n*) of corresponding points $${{\overrightarrow{{\bf{q}}}}^{k}}_{i,j}\{i=1,2;j=1,n;k=\mathrm{end} \mbox{-} \mathrm{diastole}\}$$ were extracted for the calibration purposes (Section 2.4). For CA centerlines (Section 2.6) and surface (Section 2.7) reconstruction, the total *s* vessel centerlines $${{\overrightarrow{{\rm{c}}}}^{k}}_{i,j}\{i=1,2;j=1,s;k=1,\frac{m\,for\,i=1}{p\,for\,i=2}\}$$ and its corresponding borders $${{\overrightarrow{b}}^{k}}_{i,j}\{i=1,2;j=1,2s;,k=1$$$$\frac{m\,for\,i=1}{p\,for\,i=2}\}$$ were extracted for each vessel branch; where index *i* indicates the XRA view, *j* indicates the point or centerline number, and *k* indicates the index of the XRA frame in the sequence. Both centerlines $${{\overrightarrow{{\rm{c}}}}^{k}}_{i,j}$$ and borders $${{\overrightarrow{b}}^{k}}_{i,j}$$ were defined as parametric curves, so the computation of intersections between the centerline’s normal and borders (required for the surface reconstruction) was performed automatically (Fig. 2(b)).

### Extraction of input data from XRA images

**Figure 3:**
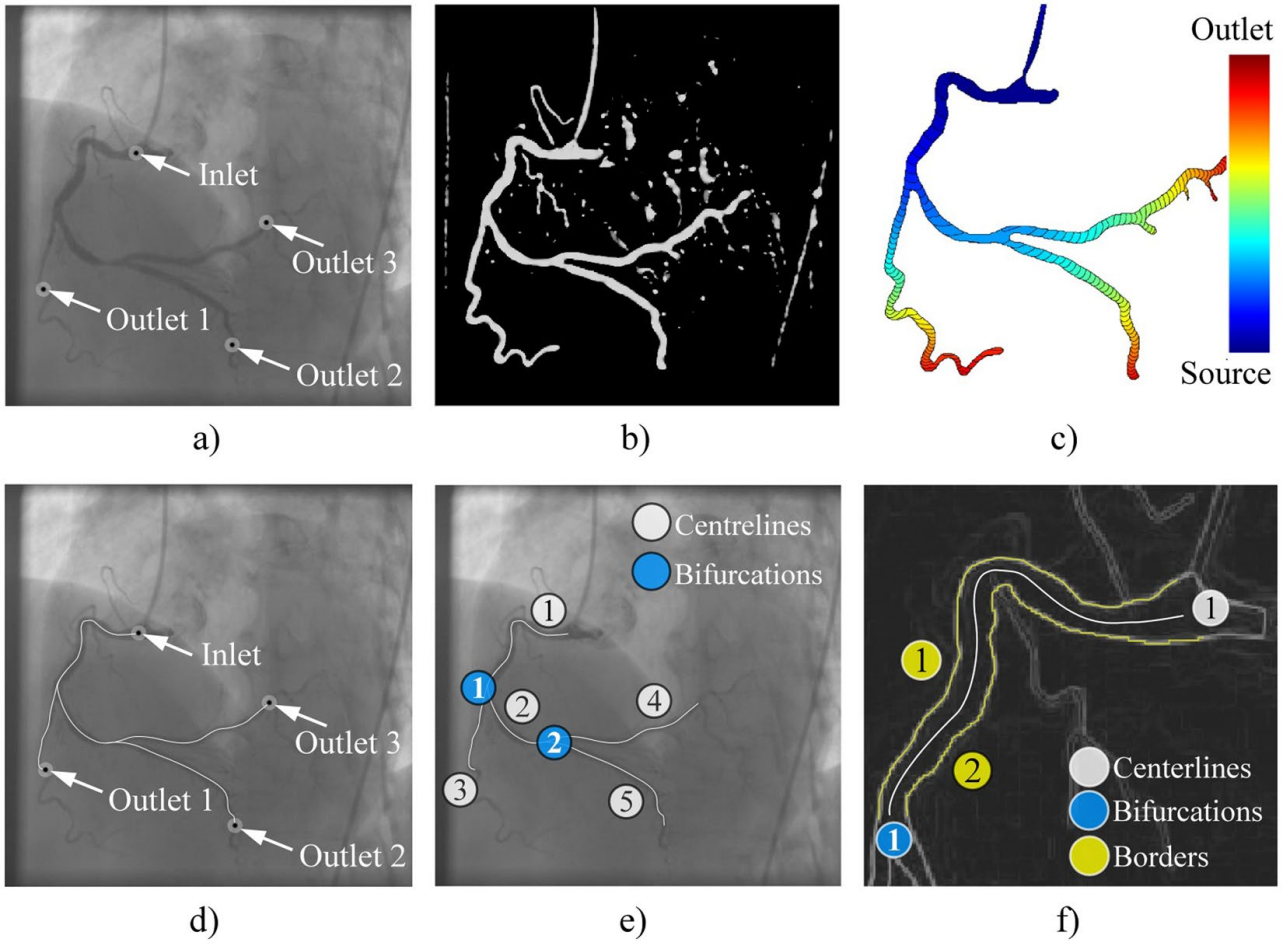
Extraction of CA centerlines and borders from XRA images. (**a**) User defined points. (**b**) Filtered image. (**c**) Wave front propagation from the CA inlet point. (**d**) Inlet-outlet paths detected from the wave-front map. (**e**) CA tree decomposed into connected branches-segments. (**f**) Detection of CA borders on the single segment using its centerline.

The procedure requires from a user to pick a start and end-point of a vessel (Fig. 3(a)), so the further extraction of its centerline and corresponding borders could be performed as described in the rest of this section.

The semi-automatic extraction of centerlines ${{\overrightarrow{{\rm{c}}}}^{k}}_{i,j}$ was performed by modeling of wave propagation over XRA image using the Eikonal equation and the Fast marching method^24,25^. Briefly, the wave was propagated starting from the user-defined start-inlet point while arrival time was stored for each pixel (Fig. 3(b,c)). Since the speed of the wave propagation depends on the image intensity, it propagates faster over the regions likely to belong to the vessel lumen and slows outside the vessel. To additionally differentiate (enhance) lumen from surrounding tissues, we pre-processed XRA images by using the Frangi filter (scale range s_min_ = 2 to s_max_ = 6, scale ratio = 0.5, λ_1_ = 0.5 and λ _2_ = 0.1) (Fig. 3(b))^26^. After the wave reach the end-point, the centerline was computed as backtrack shortest path to the source-point. In our implementation, a user first clicks on the coronary inlet (the source point) and then on multiple coronary outlets. Afterwards, the algorithm automatically computes the paths between the inlet point to the corresponding outlets (Fig. 3(d)), and decomposes the coronary tree by splitting overlapping paths to unique segments-branches ${{\overrightarrow{{\rm{c}}}}^{k}}_{i,j}$ connected at bifurcation points ${{\overrightarrow{{\bf{q}}}}^{k}}_{i,j}$ (Fig. 3(e)).

The borders ${{\overrightarrow{b}}^{k}}_{i,j}$ were detected using the extracted centerlines ${{\overrightarrow{{\rm{c}}}}^{k}}_{i,j}$. The first step was enhancing the vessel borders using the edge operator^27^ and conversion of the filtered image into the eight-node graph map of the pixel-edge values. Since the detected centerlines were parameterized, we used their inlet and outlet normals to automatically detect two border end-points (Fig. 2(b)). Potential path switching over narrowed segments was prevented by unfastening graph nodes along the vessel centerline. Finally, the borders were detected by solving the Dijkstra graph-search between the detected two borders’ end-points (Fig. 3(f))^28^.

With highly overlapping branches or missing the opaque contrast the segmentation procedure may require an additional manual editing of the obtained results.

### Mathematical model of image formation

The device positioning was expressed using these parameters: the imager pixel spacing (*κ*), the primary (*α*) and secondary (*β*) angles, the distances *d*_*sod*_ (from the patient to the X-ray source) and *d*_*sid*_ (from the X-ray source to the intensifier-plane):

1 $${\bf{M}}=[\begin{array}{cccc}\cos (\beta ) & \sin (\alpha )\sin (\beta ) & \cos (\alpha )\sin (\beta ) & 0\\ 0 & \cos (\alpha ) & -\sin (\beta ) & 0\\ -\sin (\beta ) & \sin (\alpha )\cos (\beta ) & \cos (\alpha )\cos (\beta ) & 0\\ 0 & 0 & 0 & 1\end{array}]$$

**Figure 4:**
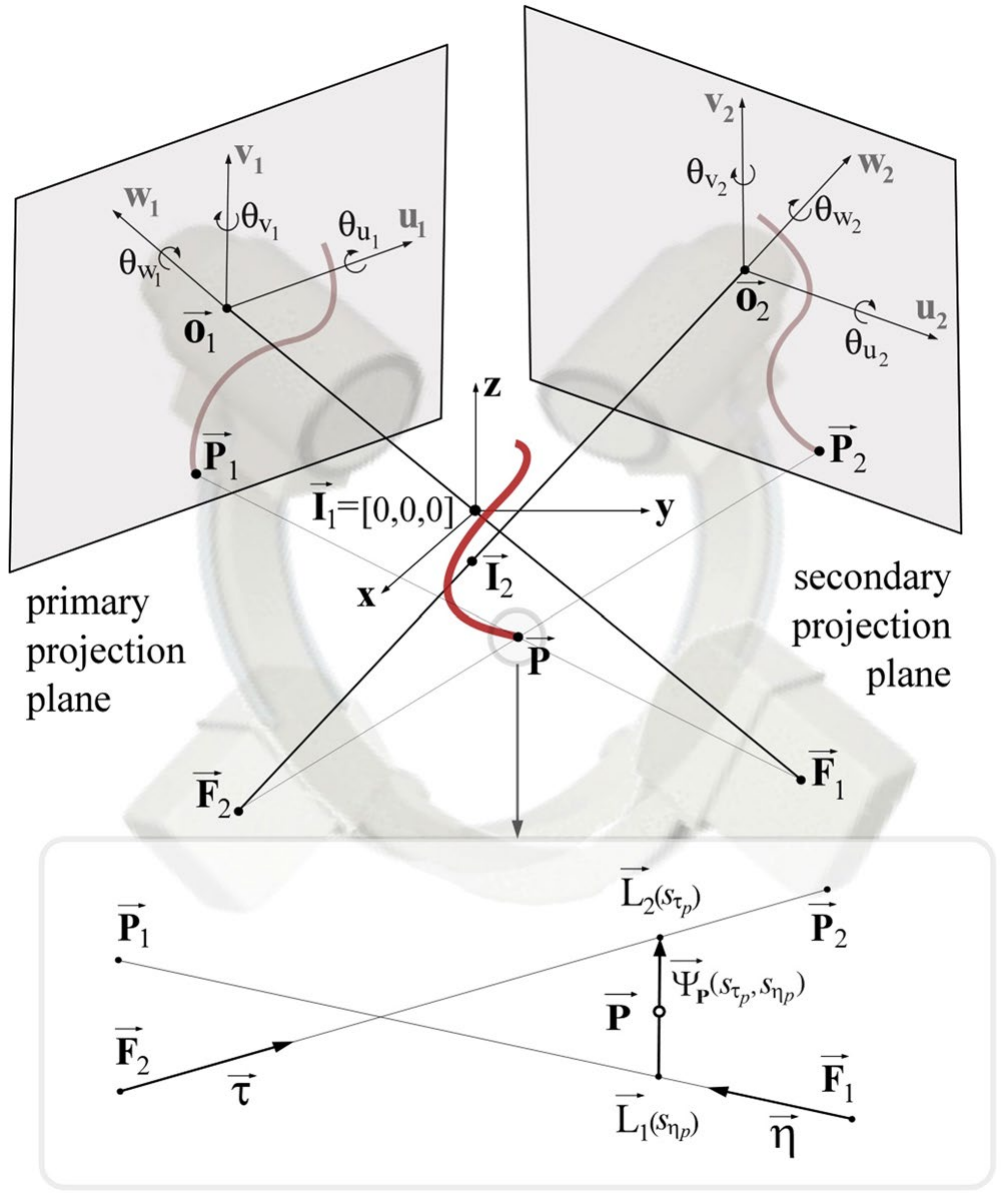
Reconstruction of a single point **P** from its two XRA projections.

Position of the X-ray source ($\overrightarrow{{\bf{F}}}$) and the origin of the projection plane ($\overrightarrow{{\bf{O}}}$) were defined as(Fig. 4):

2 $$\overrightarrow{{\bf{F}}}={\bf{M}}\cdot {[\begin{array}{cccc}0 & 0 & -{d}_{sod} & 1\end{array}]}^{{\rm{T}}}{\rm{and}}\,\overrightarrow{{\bf{O}}}={\bf{M}}\cdot {[\begin{array}{cccc}0 & 0 & {d}_{sid}-{d}_{sod} & 1\end{array}]}^{{\rm{T}}}.$$

The image-detector misalignment, caused by the nonideal mechanical response of the gantry, was defined with three rotational ${{\bf{R}}}_{{\rm{\Delta }}{\boldsymbol{\theta }}}={{\bf{R}}}_{x}(\Delta {\theta }_{x})\cdot {{\bf{R}}}_{y}(\Delta {\theta }_{y})\cdot {{\bf{R}}}_{z}(\Delta {\theta }_{z})$ and three translational $${\boldsymbol{\Delta }}\overrightarrow{{\bf{O}}}={[\begin{array}{cccc}\Delta {o}_{x} & \Delta {o}_{y} & \Delta {o}_{z} & 1\end{array}]}^{{\rm{T}}}$$ degrees of freedom. Therefore, 3D position of any 2D DICOM point $$\overrightarrow{{\bf{p}}}=[\begin{array}{cc}{p}_{u} & {p}_{v}\end{array}]=[\begin{array}{cc}{p}_{{u}_{pixel}}{\kappa }_{u} & {p}_{{v}_{pixel}}{\kappa }_{v}\end{array}]$$ was calculated as:

3 $$\overrightarrow{{\bf{P}}}=\overrightarrow{{\bf{O}}}+{{\bf{R}}}_{{\boldsymbol{\Delta }}{\boldsymbol{\theta }}}\cdot {\bf{M}}\cdot ({\boldsymbol{\Delta }}\overrightarrow{{\bf{O}}}+{[\begin{array}{cccc}{p}_{u} & {p}_{v} & 0 & 1\end{array}]}^{{\rm{T}}}).$$

Since the isocenter in the primary view ${\overrightarrow{{\bf{I}}}}_{1}$ was assumed to be fixed in the origin of the global coordinate system, the secondary view has a relative isocenter movement Δ**i**; therefore ${\overrightarrow{{\bf{I}}}}_{2}={\mathop{{\bf{I}}}\limits^{{\boldsymbol{\rightharpoonup }}}}_{1}+{\boldsymbol{\Delta }}{\bf{i}}$ and a 3D point from the secondary view was defined as $${\overrightarrow{{\bf{P}}}}_{{\rm{2}}}={\overrightarrow{{\bf{O}}}}_{{\rm{2}}}+{{\bf{R}}}_{{\boldsymbol{\Delta }}{\boldsymbol{\theta }}}\cdot {{\bf{M}}}_{2}\cdot ({\boldsymbol{\Delta }}\overrightarrow{{\bf{O}}}+{[\begin{array}{cccc}{p}_{2u} & {p}_{2v} & 0 & 1\end{array}]}^{{\rm{T}}})+{\overrightarrow{{\bf{I}}}}_{{\rm{2}}}$$. Similarly, we obtained focal points $({\overrightarrow{{\bf{F}}}}_{1},{\overrightarrow{{\bf{F}}}}_{2})$ and the projections $({\overrightarrow{{\bf{P}}}}_{1},{\overrightarrow{{\bf{P}}}}_{2}+$ of an arbitrary $\overrightarrow{{\bf{P}}}$ and used these to define two X-rays (lines) ${\overrightarrow{L}}_{1}({\overrightarrow{{\bf{F}}}}_{{\bf{1}}},{\overrightarrow{{\bf{P}}}}_{{\bf{1}}})$ and ${\vec{L}}_{2}({\vec{{\bf{F}}}}_{2},{\vec{{\bf{P}}}}_{2})$ (Fig. 4):

4 $$\begin{array}{c}\overrightarrow{L}{}_{1}({s}_{\eta })={\overrightarrow{{\bf{F}}}}_{{\bf{1}}}+{s}_{\eta }({\overrightarrow{{\bf{P}}}}_{{\bf{1}}}-{\overrightarrow{{\bf{F}}}}_{{\bf{1}}})={\overrightarrow{{\bf{F}}}}_{{\bf{1}}}+{s}_{\eta }\overrightarrow{{\boldsymbol{\eta }}}\\ {\overrightarrow{L}}_{2}({s}_{\tau })={\overrightarrow{{\bf{F}}}}_{{\bf{2}}}+{s}_{\tau }({\overrightarrow{{\bf{P}}}}_{2}-{\overrightarrow{{\bf{F}}}}_{{\bf{2}}})={\overrightarrow{{\bf{F}}}}_{{\bf{2}}}+{s}_{\tau }\overrightarrow{{\boldsymbol{\tau }}}.\end{array}$$

According to (4), the point $\overrightarrow{{\bf{P}}}$ should lie on the minimal distance between the two lines (ideally on their intersecting point). There exists a unique vector ${\overrightarrow{{\boldsymbol{\psi }}}}_{{\bf{p}}}({s}_{{\eta }_{p}},{s}_{{\tau }_{p}})$ defined by two points ${\overrightarrow{{\rm{L}}}}_{1}({s}_{{\eta }_{p}})$ and ${\overrightarrow{{\rm{L}}}}_{2}({s}_{{\tau }_{p}})$ that is normal to the direction of both lines. By solving these equations for the parameters ${s}_{{\eta }_{p}}$ and ${s}_{{\tau }_{p}}$, the point $\overrightarrow{{\bf{P}}}$ was calculated as:

5 $$\overrightarrow{{\bf{P}}}=({\overrightarrow{{\rm{L}}}}_{1}({s}_{{\eta }_{p}})+{\overrightarrow{{\rm{L}}}}_{2}({s}_{{\tau }_{p}}))/2.$$

### Device calibration using two XRA end-diastole frames

If the primary view was posterior-anterior (*α*_1_ = 0, *β*_1_ = 0), a genetic algorithm (GA)^29^ was used to identify the 11 device parameters ${\bf{X}}{\bf{A}}\{{\alpha }_{2},{\beta }_{2},{\boldsymbol{\Delta }}{\boldsymbol{\theta }},{\boldsymbol{\Delta }}\overrightarrow{{\bf{O}}},{\boldsymbol{\Delta }}\overrightarrow{{\bf{i}}}\}$ by minimizing the 2D mean square error (MSE) defined as:

6 $$\begin{array}{c}{\rm{\arg }}\,{\rm{\min }}\,{\rm{F}}({\bf{X}}{\bf{A}})\\ \mathop{{\bf{X}}{\bf{A}}}\limits^{\ast }\end{array}=\frac{\sum _{i=1}^{2}\sum _{j=1}^{n}{|{\overrightarrow{{\bf{q}}}}_{i,j}-{\overrightarrow{{\bf{q}}}}_{i,j}({\overrightarrow{{\bf{Q}}}}_{j})|}^{2}}{n},$$

where *i* indicates XRA projection number, *j* indicates point number (from total *n* samples), ${\overrightarrow{{\bf{q}}}}_{i,j}$ is the sampled point from *i*-th XRA view, and ${\overrightarrow{{\bf{q}}}}_{i,j}({\overrightarrow{{\bf{Q}}}}_{j})$ represents a ray-tracing projection of the obtained 3D point ${\overrightarrow{{\bf{Q}}}}_{j}$ back onto the XRA detector in the *i*-th view. When reconstructing a complex coronary tree, the sampled points ${\overrightarrow{{\bf{q}}}}_{i,j}$ were the bifurcation points; otherwise, when reconstructing a single branch we used its end-points (Dataset III-D). Therefore, the calibration algorithm requires from a user to pick a single corresponding pair of frames at end-diastole and it returns optimized XRA parameters $\mathop{{\bf{X}}{\bf{A}}}\limits^{\ast }$ and the calibration error $\mathop{{\varepsilon }_{{\rm{\min }}}}\limits^{\ast }={\rm{F}}(\mathop{{\bf{X}}{\bf{A}}}\limits^{\ast })$.

### Partial matching of frames acquired from two XRA projections

By using breath-hold acquisitions, the gating problem could be reduced to the temporal alignment of XRA frames. However, due to the shifted cardiac phases and different lengths of acquisitions, it remains a challenging task, which usually demands an expert user interaction. The proposed procedure based on the optimal subsequence bijection^30–32^ aims to automatize this task. Briefly, if the series of *m* frames from the primary view **a** = (*a*_1_, …, *a*_*m*_) and *p* frames from the secondary view **b** = (*b*_1_, …, *b*_*p*_) are available, it computes the subsequences ${\bf{a}}\text{'}$ of **a** and ${\bf{b}}\text{'}$ of **b** (so that ${\bf{a}}\text{'}$ best matches ${\bf{b}}\text{'}$). For each vertex (*q* = 1 … *m*,*w* = 1 … *p*), if $q < k\wedge w < l$, the edge-cost matrix was computed as:

7 $${\bf{D}}((q,w)(k,l))=\sqrt{{(k-q-1)}^{2}+{(l-w-1)}^{2}}\xi ({\bf{a}},{\bf{b}})+{\rm{\delta }}({a}_{k},{{\rm{b}}}_{l}).$$

Or set to ∞. The optimal frame correspondence was obtained by optimizing the trade-off between the dissimilarity of ${\bf{a}}\text{'}$ and ${\bf{b}}\text{'}$ while the penalty for skipping elements from **a** and **b** was defined as:

8 $$\xi ({\bf{a}},{\bf{b}})=\mathop{{\rm{\min }}}\limits_{{\rm{q}}}(\mathop{{\rm{\min }}}\limits_{{\rm{w}}}({\rm{\delta }}({a}_{q},{b}_{w})))+\mathop{{\rm{std}}}\limits_{{\rm{q}}}(\mathop{{\rm{\min }}}\limits_{{\rm{w}}}({\rm{\delta }}({a}_{q},{b}_{w}))).$$

Since *n* points were sampled from the two XRA views, the matching arrays were defined as series of these points ${\bf{a}}={\overrightarrow{{\bf{q}}}}_{i,j}^{k}\{i=1;j=\mathrm{1...}n;k=\mathrm{1...}m\}$ and ${\bf{b}}={\overrightarrow{{\bf{q}}}}_{i,j}^{k}\{i=2;j=\mathrm{1...}n;k=\mathrm{1...}p\}$:

9 $${\rm{\delta }}({a}_{k},{{\rm{b}}}_{l})=\frac{\sum _{i=1}^{2}\sum _{j=1}^{n}{|{\overrightarrow{{\bf{q}}}}_{i,j}-{\overrightarrow{{\bf{q}}}}_{i,j}({\overrightarrow{{\bf{Q}}}}_{j})|}^{2}}{n}.$$

The dissimilarity function δ was calculated using the function ${\rm{F}}(\mathop{{\bf{X}}{\bf{A}}}\limits^{\ast })$ from (6) for each pair of candidates (*a*_*k*_, b_*l*_). The obtained result was the array of optimal correspondences (pairs) **CF** between the series of XRA frames, suitable for the further sequential reconstruction of paired CA frames.

### Vessel centerline reconstruction

**Figure 5:**
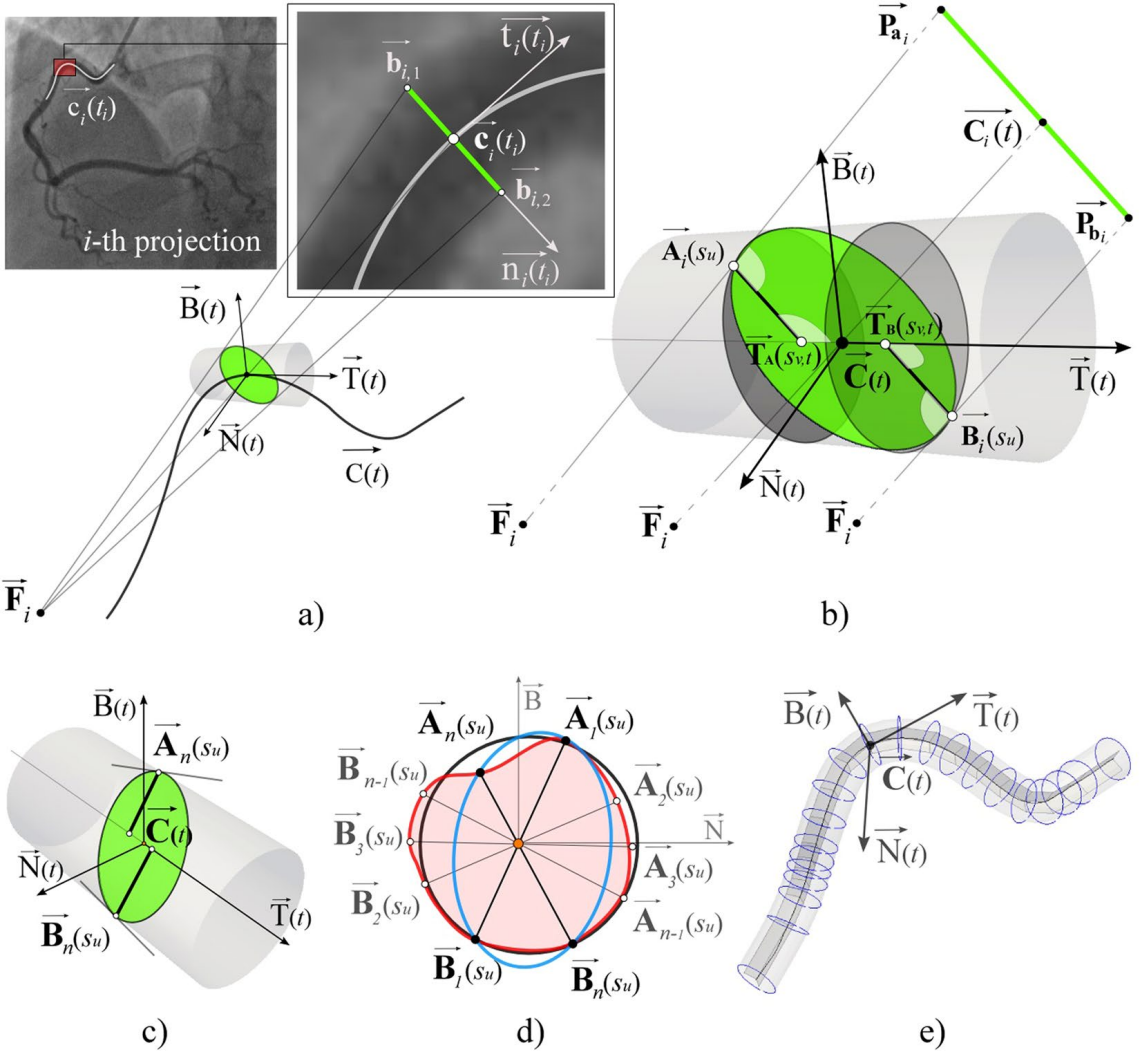
Reconstruction of CA lumen surface from its XRA projections. (**a**) Parametric centreline curve C(t) and Frenet–Serret trihedron defined at the centreline point **C**. (**b**) Reconstruction of two points (**A**_i_ and **B**_i_) on the lumen border. (**c**) Detecting **A**_n_ and **B**_n_ from n-th view. (**d**) Combining points **A** and **B** for fitting circle (black) or ellipse (blue) when two XRA views are acquired and polygonal approximation (red) from multiple XRA views. (**e**) Obtained vessel cross-sections oriented along their centreline C(t).

Centerline reconstruction requires knowing point correspondence across XRA views. While methods based on the matching of epipolar points using dynamic programing (DP) may fail producing extra point-pairs, both DP and active contours methods are sensitive on over/under estimation of centerlines during the segmentation. Considering an arbitrary *j*-th vessel centerline from an arbitrary *k*-th corresponding pair of frames in the list **CF**, the proposed procedure aims to avoid these flaws by partial matching (described in section 2.5) of centerline projections (${\overrightarrow{{\rm{c}}}}_{1,j}^{{\rm{k}}}$ and ${\overrightarrow{{\rm{c}}}}_{2,j}^{{\rm{k}}}$). If the centerline ${\overrightarrow{{\rm{c}}}}_{1,j}^{{\rm{k}}}$ is interpolated with g points and centerline ${\overrightarrow{{\rm{c}}}}_{2,j}^{{\rm{k}}}$ is interpolated with h points, the penalty for skipping points from the two point series ${\bf{a}}={\overrightarrow{{\bf{c}}}}_{i,j}\{i=1;j=1\,\mathrm{...}\,g\}$ and ${\bf{b}}={\overrightarrow{{\bf{c}}}}_{i,j}\{i=2;j=1\,\mathrm{...}\,h\}$ was set to:

10 $$\xi ({\bf{a}},{\bf{b}})=\sqrt{2}\mathop{{\varepsilon }_{{\rm{\min }}}}\limits^{\ast },$$

where $\mathop{{\varepsilon }_{{\rm{\min }}}}\limits^{\ast }$ is the calibration error calculated using (6). The values in the cost matrix were calculated as:

11 $${\rm{\delta }}(r,t)={|{\overrightarrow{{\bf{c}}}}_{{\rm{1}},r}-{\overrightarrow{{\bf{c}}}}_{1,r}({\overrightarrow{{\bf{C}}}}_{rt})|}^{2}+{|{\overrightarrow{{\bf{c}}}}_{2,t}-{\overrightarrow{{\bf{c}}}}_{2,t}({\overrightarrow{{\bf{C}}}}_{rt})|}^{2}$$

if the condition $1\le r\le g\wedge 1\le t\le h$ was satisfied; or set to be 0 if $(r=0\wedge t=0)\vee (r=g+1\vee t=h+1)$ and ∞ otherwise. In equation (11), ${\overrightarrow{{\bf{C}}}}_{rt}$ is the 3D point reconstructed by pairing *r*-th (${\overrightarrow{{\bf{c}}}}_{1,r}$) point from the primary view and *t*-th point (${\overrightarrow{{\bf{c}}}}_{2,t}$) from the secondary view, whereas ${\overrightarrow{{\bf{c}}}}_{1,r}({\overrightarrow{{\bf{C}}}}_{rt})$ and ${\overrightarrow{{\bf{c}}}}_{2,t}({\overrightarrow{{\bf{C}}}}_{rt})$ are the ray-tracing projections of ${\overrightarrow{{\bf{C}}}}_{rt}$ back on the positioned intensifier. Finally, the resulting-corresponding series of points $\mathop{{\bf{C}}{\bf{P}}}\limits^{\ast }$ were used to define the nonuniform B-spline curve $\overrightarrow{{\rm{C}}}(t)$ shown in Fig. 5(a)^33^:

12 $$\overrightarrow{{\rm{C}}}(t)=\sum _{i=1}^{q}{\mathop{{\bf{C}}{\bf{P}}}\limits^{\ast }}_{i}^{h}{{\rm{N}}}_{i,k}(t),\,{\rm{where}}\,0\le t\le 1\,{\rm{and}}\,2\le k\le i+1.$$

In equation (12), $\mathop{{\bf{C}}{\bf{P}}}\limits^{\ast }$ are the 4D homogenous control points and N_i,k_ are *k*-th order basis functions. The basis functions were calculated according to the Cox–de Boor recursive algorithm^33^:

13 $$\begin{array}{c}{{\rm{N}}}_{i,1}(t)=\{\begin{array}{l}1\,{\rm{if}}\,{s}_{i}\le t < {s}_{i+1}\\ 0\,{\rm{otherwise}}\end{array}\}\\ {{\rm{N}}}_{i,k}(t)=\frac{(t\,-\,{s}_{i}){N}_{i,k-1}(t)}{{s}_{i+k-1}\,-\,{s}_{i}}+\frac{({s}_{i+k}\,-\,t){N}_{i+1,k-1}(t)}{{s}_{i+k}\,-\,{s}_{i+1}},\end{array}$$

where **s** represents the knot vector calculated using the Chord length algorithm.

### 3D modeling of a single coronary branch surface

An arbitrary *j*-th coronary artery (obtained from the *k*-th corresponding pair of frames in the list **CF**) was modeled as a tube-like surface defined with cross-sections positioned along the parameterized centerline $\overrightarrow{{\rm{C}}}(t)={\overrightarrow{{\rm{C}}}}_{j}^{k}(t)$ (Fig. 5). Using the Frenet–Serret formulas, the trihedron at the point $\overrightarrow{{\bf{C}}}(t)$ was defined via the curve’s tangent $\overrightarrow{{\rm{T}}}(t)$, normal $\overrightarrow{{\rm{N}}}(t)$ and binormal $\overrightarrow{{\rm{B}}}(t)$ (Fig. 5(a))^33^. The corresponding centerline point on *i*-th view was estimated as ${\overrightarrow{{\bf{c}}}}_{i}({t}_{i})$ using the procedure described in the section above (note that *t* ≠ *t*_*i*_). Therefore, the vessel cross-section on the *i*-th XRA frame was defined at the point ${\overrightarrow{{\bf{c}}}}_{i}({t}_{i})$, while its tangent ${\overrightarrow{{\rm{t}}}}_{i}({t}_{i})$ and normal ${\overrightarrow{{\rm{n}}}}_{i}({t}_{i})$ were computed from the parametric 2D curve ${\overrightarrow{{\rm{c}}}}_{{\rm{i}}}({t}_{i})$. Considering that, we first computed the intersections of the 2D normal ${\overrightarrow{{\rm{n}}}}_{i}({t}_{i})$ with the corresponding vessel borders ${\overrightarrow{{\rm{b}}}}_{i,1}({t}_{i})$ and ${\overrightarrow{{\rm{b}}}}_{i,2}({t}_{i})$. By using (3) obtained border-normal intersecting points ${\overrightarrow{{\bf{b}}}}_{i,1}$ and ${\overrightarrow{{\bf{b}}}}_{i,2}$ were positioned in 3D and defined as ${\overrightarrow{{\bf{P}}}}_{a,i}$ and ${\overrightarrow{{\bf{P}}}}_{b,i}$, respectively (Fig. 5(b)). The lines (X-rays) from (4) were: ${\mathop{{\rm{L}}}\limits^{\rightharpoonup }}_{1}({\overrightarrow{{\rm{F}}}}_{i},{\overrightarrow{{\bf{P}}}}_{{{\bf{a}}}_{i}})$ defined by the X-ray source point ${\overrightarrow{{\rm{F}}}}_{i}$ and point ${\overrightarrow{{\bf{P}}}}_{{{\bf{a}}}_{i}}$ and the second line ${\mathop{{\rm{L}}}\limits^{\rightharpoonup }}_{2}(\overrightarrow{{\bf{C}}}(t),\overrightarrow{{\rm{T}}}(t))$ was defined by the point $\overrightarrow{{\bf{C}}}(t)$ and direction vector $\overrightarrow{{\rm{T}}}(t)$. By solving (4), the point where the ray ${\overrightarrow{{\rm{L}}}}_{2}$ is tangent to the vessel surface was obtained and defined as ${\overrightarrow{{\bf{A}}}}_{i}({s}_{u},t)={\overrightarrow{{\rm{L}}}}_{2}({s}_{{u}_{p}})$. Finally, the position of a single point for defining one patch (vessel cross-section) was obtained by projecting ${\overrightarrow{{\bf{A}}}}_{i}({s}_{u},t)$ onto the trihedron normal-binormal plane. By repeating the same procedure for ${\overrightarrow{{\bf{b}}}}_{i,2}\to {\overrightarrow{{\bf{P}}}}_{b,i}$, the second point ${\overrightarrow{{\bf{B}}}}_{i}({s}_{u},t)$ was obtained (Fig. 5(c,d)).

From the two XRA views, four points could be obtained per patch (Fig. 5(d) black) which will fit a circle^34^. By repeating the procedure for multiple views, either an elliptic (Fig. 5(d) blue)^35^ or a more accurate polygonal representation of the vessel patch could be obtained as shown in Fig. 5(d) (red). Regularity of points was ensured by converting them into the polar coordinates in normal-binormal plane and sorting in circular direction. Finally, the obtained 3D patches were used for NURBS surface representation and structured quadrilateral or hexahedral meshing of CA lumen surface (Fig. 5(e))^33,36^:

14 $$\overrightarrow{S}(u,v)=\sum _{i=1}^{q}\sum _{j=1}^{w}{{\overrightarrow{{\bf{B}}}}^{h}}_{i,j}{{\rm{N}}}_{i,k}(u){{\rm{M}}}_{j,l}(v),\,{\rm{where}}\,0\le u\le 1\,{\rm{and}}\,0\le v\le 1,$$

where ${{\overrightarrow{{\bf{B}}}}^{h}}_{i,j}$ are the 4D homogenous points of the control net polygon, and N_*i,k*_ and M_*j,l*_ are basis functions of order *k* and *l*, respectively, calculated according to equation (13). Since the control net must be topologically rectangular, we interpolated each of the *q* patches with *w* points (twisting of patches along the branches was avoided by sorting the control points in the circular direction of the normal-binormal plane).

### NURBS-based meshing of a single coronary branch

**Figure 6:**
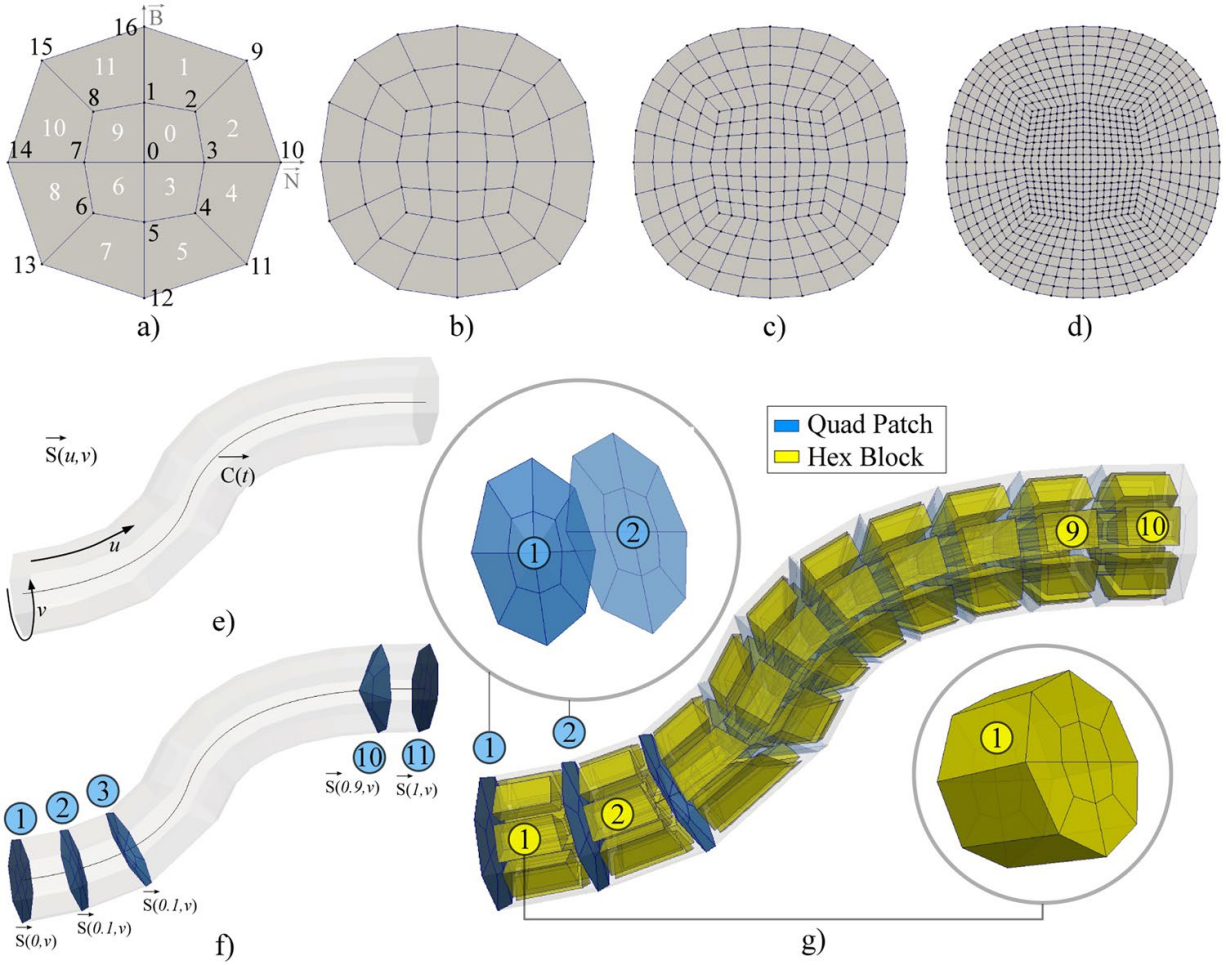
Meshing of a single CA branch. (**a–d**) Examples of the patch (CA cross-section) obtained by increasing the number of circular subdivisions(*v*). (**e**) CA surface $\overrightarrow{S}(u,v)$ and centerline $\overrightarrow{{\rm{C}}}(t)$ (during the hex-meshing u = t). (**f**) Generation of quadrilateral patches over the CA. (**g**) Generation of the hex-blocks using successive quad-patches (there are 10 subdivisions in the longitudinal-*u* and 8 subdivisions in the circular-*v* direction).

Discretization of a single CA branch was performed using the previously obtained parameterized vessel centerline $\overrightarrow{{\rm{C}}}(t)$ and surface $\overrightarrow{S}(u,v)$, enabling us to vary the mesh density in both longitudinal *u* and circular *v* directions (Fig. 6). Briefly, the procedure decomposes CA volume into connected hexahedra elements blocks, which were composed of quadrilateral patches (vessel cross sections). The supported quadrilateral patch is given on Fig. 6(a–d). Considering Fig. 6(a), which includes the enumeration of nodes and quadrilateral elements, we will introduce our approach for the patch generation assuming that it is positioned at an arbitrary position along the CA branch. The patch pattern is generated using the point in the patch centre and octagon that maps to the arbitrary shaped outer contour. It is symmetric in two directions (normal-binormal), which enables us efficient representation of connected CA branches (see the next section for detailed description of meshing complex CA trees).

Node with id 0 represents the central point and it was obtained by interpolating the vessel centreline $\overrightarrow{{\rm{C}}}(t)$. In the example shown on Fig. 6(b), the number of circular subdivisions was set to 8 – so that the outer-nodes (id = 9 … 16) were obtained by interpolating the surface in circular (v) direction $\overrightarrow{S}(u=t,v=0:1/8:1)$. The octagon-nodes (id = 1 … 8) were assumed to be at 45% distance between the central-node (id = 0) and the corresponding outer-nodes (id = 9 … 16). Following Fig. 6(a), the patch is subdivided into the quadrants – so that loop could be used to discretize the octagon and map the octagon-edges to the outer-edges. Assuming that one increased the circular subdivision to 16 (Fig. 6(b)), the procedure will interpolate 16 outer-nodes $\overrightarrow{S}(t,v=0:1/16:1)$ and 16 octagon-nodes while the rest of the procedure remains the same (Fig. 6(c,d)).Generation of the patches along the CA branch is shown on Fig. 6(f) (the longitudinal subdivision was set to 10 while the circular subdivision was set to 8). A single block of hexahedral elements was obtained by combining two successive quadrilateral patches, where their composing quadrilaterals were assumed to be the hexahedra-block faces (Fig. 6(g)).

### 3D modeling of complex CA trees as connected branches

**Figure 7:**
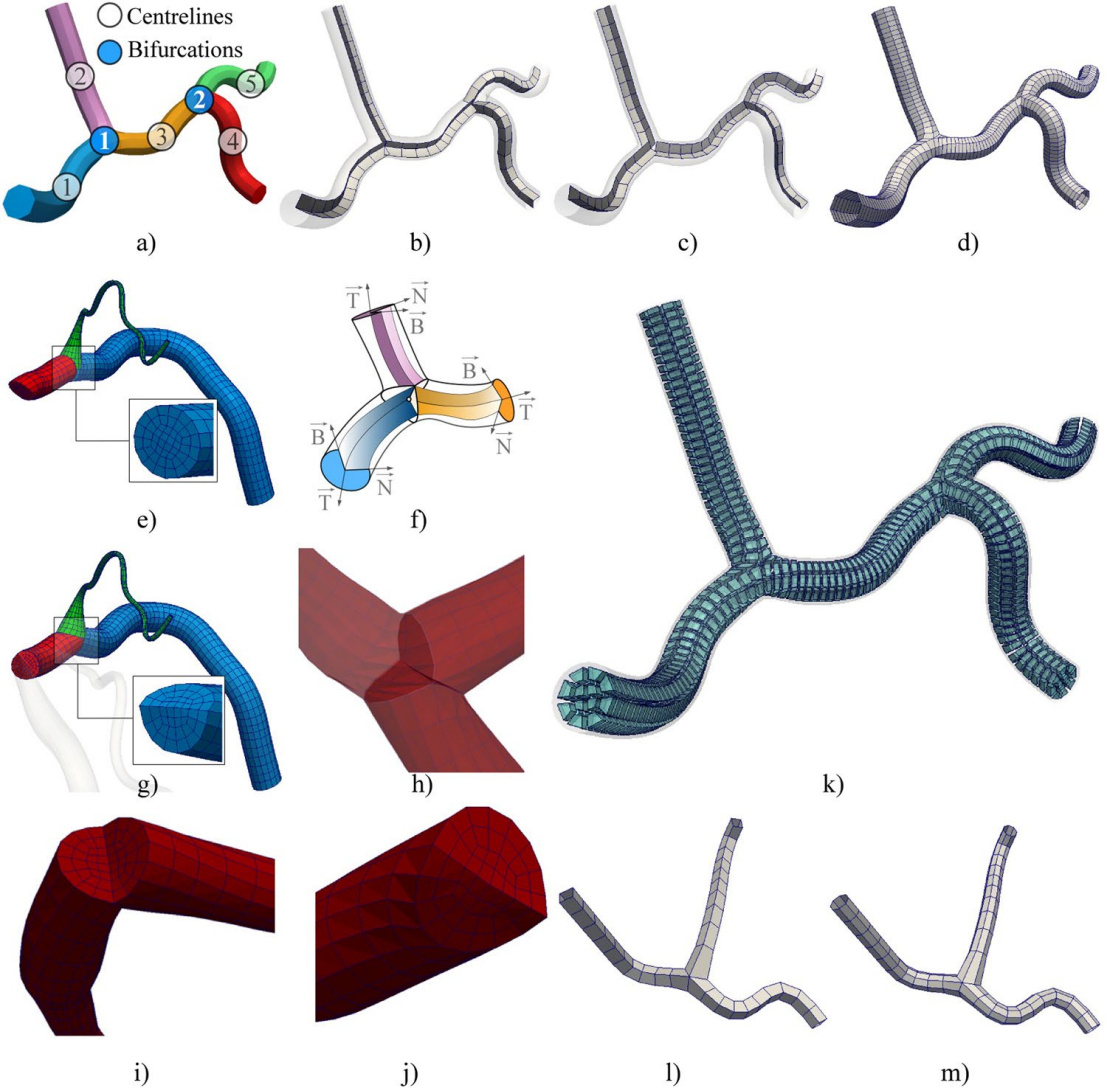
Structured meshing of complex CA trees. (**a**) Decomposed CA tree. (**b**) Default orientation of Frenet–Serret trihedrons along branches. (**c**) Regularized-smoothed orientations of the trihedrons. (**d**) Resulting surface mesh. (**e–g**) Bifurcation trimming and branches merging. (**h–j**) Sample trimmed bifurcation. (**k**) Resulting hexahedra mesh. (**l–m**) Examples of bifurcation surfaces obtained by decreasing the number of circular subdivisions (to 4 and 8).

Coronary trees were modeled as a directed graph, where CA branches (tubular structures) represent graph nodes and bifurcation points represent the graph edges (a single bifurcation has one input and two output branches-nodes) (Fig. 7(a)). After the vessel centerlines were reconstructed independently (Section 2.6), the orientations of their trihedrons were smoothed according to the end-trihedron of the input branch for each bifurcation following Fig. 7(b,c,d). The procedure from Section 2.7 was performed for every branch in the list. For each bifurcation in the series, the bifurcation sides of the corresponding branches were trimmed following the pattern illustrated in Fig. 7(e–j)^37–39^. Trimming planes (Fig. 7(f,h)) were assumed to be symmetric to the directions of its corresponding branches. The trimming process consists of modifying the NURBS control points using the following approach. First, the trim-planes were used to remove end-patches, and afterwards, we added one new shared contour. The shared contour was obtained by averaging projections of nearest removed contours of two connected branches onto the trim-planes. On this way, elements continuity between connected branches was ensured during the subsequent quadrilateral or hexahedra meshing of complex CA trees. Finally, the mesh of the complete CA tree was obtained by merging meshes of the independently processed branches (the graph nodes – see S1 - video) using the procedure described in Section 2.8. Figure 7(k) shows results of meshing our sample CA tree composed of two bifurcations, while Fig. 7(l,m) demonstrate how a single bifurcation mesh changes with decreasing the number of circular subdivisions.

### Acquisition of imaging data sets - description and purpose

To assess the performance and accuracy of the proposed method, we designed series of experiments that represent increasingly realistic and challenging scenarios ranging from digital and physical phantoms to real clinical data sets.

**Figure 8:**
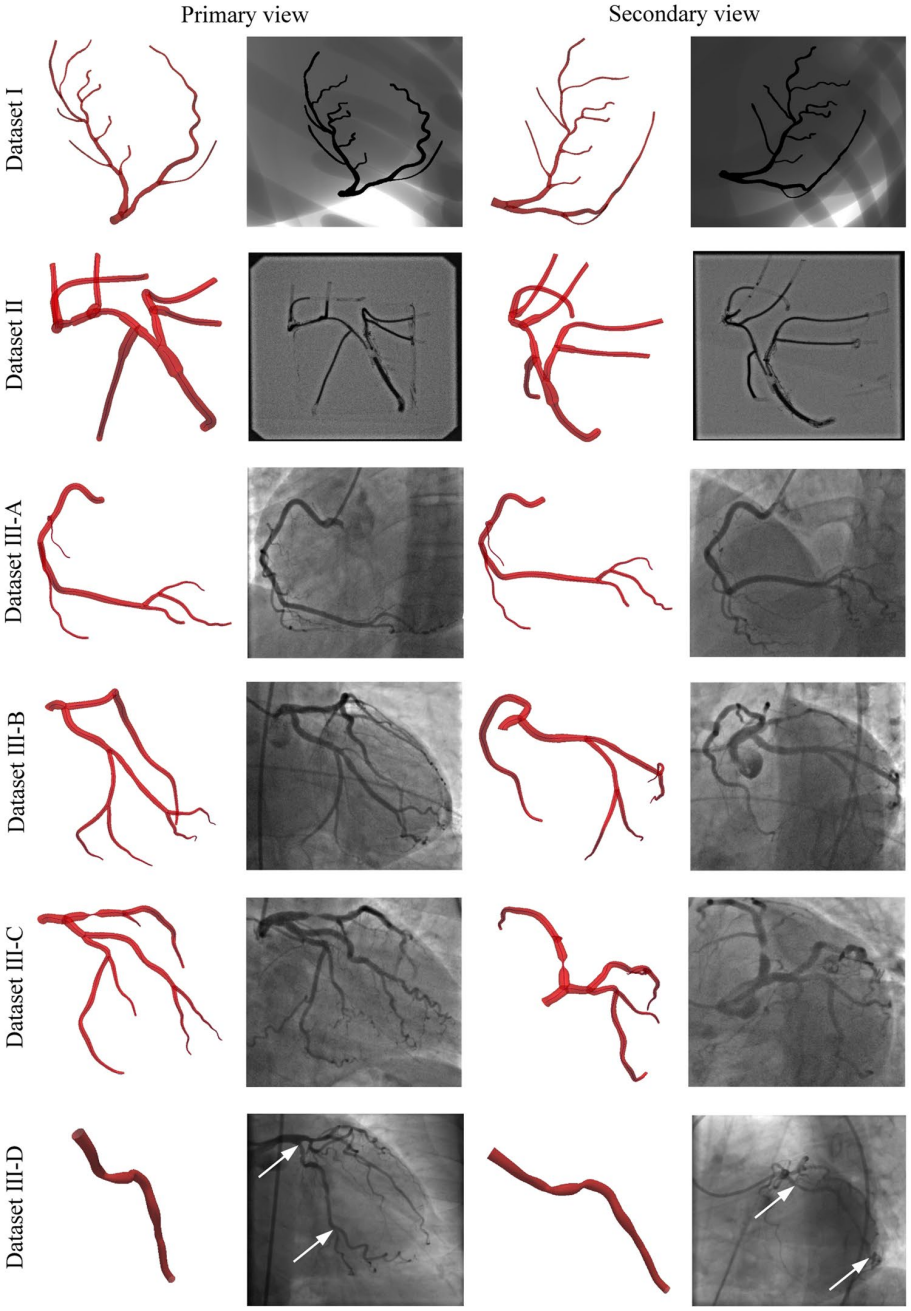
Input images and reconstructions of the considered data sets.

Dataset I is a realistic digital phantom based on the 4D XCAT^40^ (Fig. 8). The image resolution was set to 960 × 960 pixels, the pixel spacing was set to 0.184 mm, the frame rate was set to 30 fps, and the cardiac and respiratory cycles were set to 1 s and 5 s, respectively. Since the ground truth was known *a priori*, the purpose of Dataset I was to study the influence of variations in lumen narrowing, XRA angulations and phase shifting of the acquired frames on the results.

Dataset II is a physical static phantom made of connected rubber tubing filled with the iodinated contrast agent (Fig. 8). The data were collected using the GE Innova 2100-IQ (GE Healthcare, Waukesha, WI) angiography system with a flat-panel detector (1024 × 1024 pixel resolution at 15 fps and a pixel size of 0.287 mm). The referent geometry of the phantom was obtained using multislice 64-slice CT (MSCT) Toshiba Aquilion System (Toshiba Medical Systems, Otawara, Japan). A total of 467 slices with thickness of 0.35 mm and 512 × 512 pixels resolution were acquired. Therefore, the purpose of Dataset II was to compare our results with geometry obtained from MSCT scans.

Dataset III represents elective CAs (Fig. 8 shows four of 20 considered – 15 RCAs and 5 LCAs) with CAD and stable angina pectoris acquired. All the acquisitions were performed using a routine protocol and the same X-ray imaging equipment as used for the phantom Dataset II. Data of the patient who underwent both XRA and MSCT were used to additionally compare XRA results with geometry obtained from MSCT scans under the clinical conditions. The Dataset III represents data that had been routinely collected during patients’ examination in Clinical Center Kragujevac, Kragujevac, Serbia. The data were selected from the already existing clinic’s database (post hoc). The selection was approved by the Clinical Center ethical committee and was not related with the therapeutic outcomes of the patients that signed informed consent.

### Data Availability

The datasets generated and analyzed during the study are publicly available in the Dropbox repository (link: https://www.dropbox.com/sh/ytn5g5idp507j4k/AAAjQOe-Zu9kZGiSH-Zxl4Boa?dl = 0). Folders contain the referent geometries, input images (DICOMs) and obtained geometries with supporting video clips. Data are organized in three folders (see section 2.10 for the detailed description):

- Dataset I - static phantom (all presented data and results are provided with no restrictions).
- Dataset II - digital phantom (digital phantoms images were generated by XCAT software^40^ available online at https://olv.duke.edu/technologies/4d-extended-cardiac-torso-xcat-phantom-version-2-0/).
- Dataset III - clinical data (sample anonymized data are provided online). The rest of data are available from the corresponding author upon reasonable request (due to ethical reasons and patient privacy protection).

## Results and Discussion

### Adopted accuracy indicators

The validation was split into several subsections to assess each reconstruction step individually. Despite extensive progress during the past decades, no procedure for measuring accuracy of CA reconstructions from XRA has been standardized so far. Instead, a few indicators were frequently used including the measurement of: difference between ground truth and obtained vessel diameters along the branch^41^, branches length^42^, bifurcation angle^18^, and mean square error (MSE) of the projection^9^. Since the proposed framework integrates several steps and aims to deliver surfaces, we preferred measuring the deviation of the obtained CA surface geometry from its corresponding ground truth. The surface deviation was used to expresses the overall accuracy, since it accounts indicators used in the literature that studied separate reconstruction steps. After aligning two surfaces, the deviation was calculated as the closest point normal to the surface for each vertex in the mesh. This metric does not assume point correspondences between two surfaces, but in contrast to the conventional Hausdorff distance it expresses the sign of the deviation. We found beneficial the possibility to visually interpret over/under estimated regions (the negative deviation sign indicates omitted narrowing, while the positive deviation indicates overestimated regions). The quantitative error analysis also included measuring of mean, max, min and standard deviation for the surface deviation and estimation of the correlation coefficients for the centerlines.

### Device calibration

Considering all three datasets, the calibration procedure was assessed by studying the sensitivity on inter-observer variability (six clinicians) and number of sampled points on the results accuracy. The procedure was compared versus two alternative approaches from literature: the first (A1) assumes that the C-arm is positioned by applying the primary rotation *α* followed by the secondary rotation *β*; distances (*d*_*SOD*_, *d*_*SID*_), intensifier in-plane motions and intrinsic parameters were accounted via projection matrix following literature^7,8,14^. The second approach (A2) decreases the complexity by fixing one projection so the second view is determined with three rotations around the x, y and z axes; while the projection matrix accounted for the intensifier in-plane skewness and movement of the patient table^9^.

**Figure 9:**
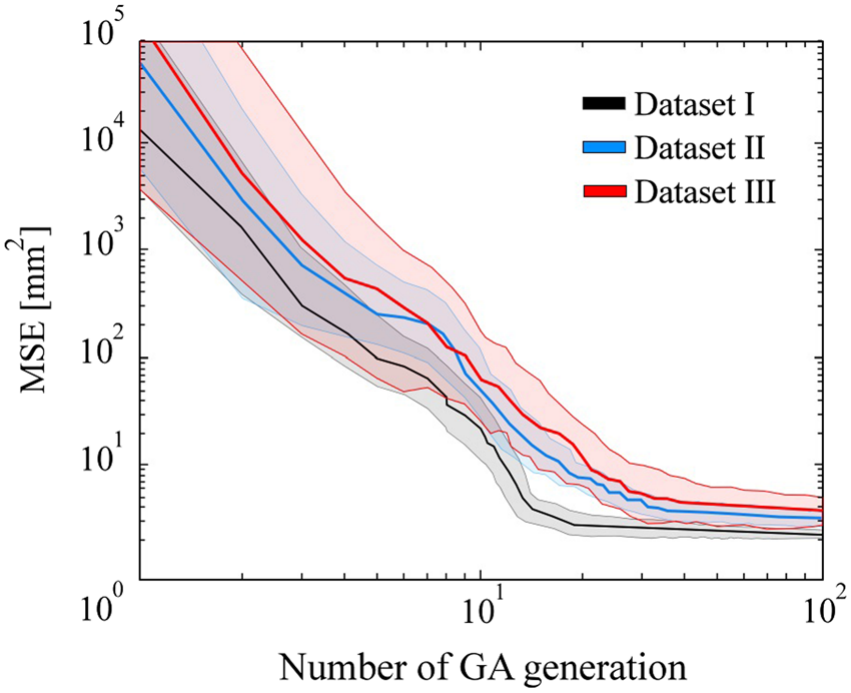
Analysis of inter-observer variability (six users) during the calibration (average MSE and the corresponding deviation over the GA iterations).

The obtained results are given in Fig. 9 and Table 2. Compared to the alternatives that compensate XRA gantry misalignments by including various parameters into the projection matrix, the proposed model based on ray-casting accounts all misalignments reported separately in literature (misalignments of the isofocus, patient table and intensifier). Therefore, it keeps the number of optimization parameters low while it eases the further reconstruction of CA surface. From Fig. 9 it may be found that the proposed approach is not sensitive to inter-observer variability. Results from Table 2 showed that the robust genetic optimizer is competitive (in calibration error and resulting surface deviation) with alternative state-of-the-art approaches based on using the Levenberg-Marquardt optimizer (the values in Table 2 correspond to the end-diastole moment used for the calibration)^7,9,15^.

**Table 2:** Influence of number of sampled points on the results.

Data set	Approach	Single branch (2 sampled points)		Coronary tree (3 + branches; 4–12 points)	
		Average calibration MSE [mm^2^]	Average surface deviation [mm]	Average calibration MSE [mm^2^]	Average surface deviation [mm]
I	Proposed	1.917	0.271/−0.264	1.282	0.233/−0.202
	A1^7,14^	2.311	0.304/−0.310	1.784	0.301/−0.285
	A2^9^	2.285	0.296/−0.388	1.693	0.307/−0.296
II	Proposed	2.574	0.536/−0.538	2.088	0.519/−0.527
	A1^7,14^	3.056	0.620/−0.609	2.752	0.601/−0.579
	A2^9^	2.808	0.624/−0.614	2.639	0.612/−0.620
III	Proposed	3.259	(N.A)	2.532	(N.A)
	A1^7,14^	4.073	(N.A)	3.159	(N.A)
	A2^9^	3.610	(N.A)	2.894	(N.A)

### Temporal synchronization of XRA frames

**Figure 10:**
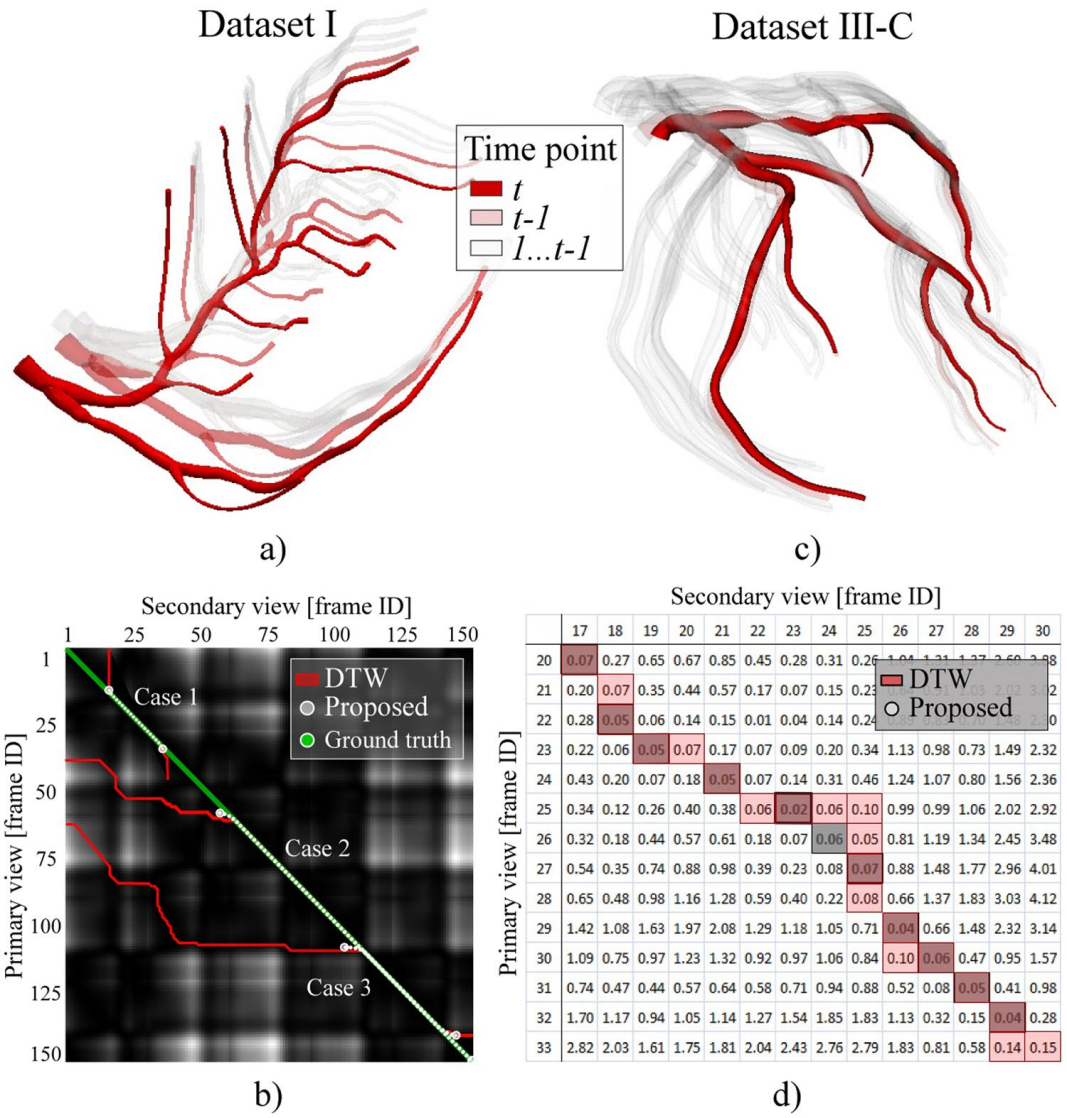
Frame synchronization. (**a,b**) Data set I. (**a**) Obtained 3D + t surface. (**b**) Frame-correspondence matrix with the obtained correspondence paths for the three cases of mismatching the lengths and phases between XRA acquisitions. (**c,d**) Dataset III-C. (**c**) Obtained 3D + t surface. (**d**) Frame-correspondence matrix with the obtained frame-correspondence.

Temporal synchronization of XRA frames was assessed by using the clinical data Dataset III-C and 4D phantom Dataset I (correspondences were known a priori). To compare with the state of the art sequential approaches based dynamic programming^12–14,16,19^, we provided both DTW and the proposed procedure with four test cases assuming various mismatching of lengths and phases between the acquired XRA frame sequences (Table 3, Fig. 10b,d).

**Table 3:** Influence of XRA frames phase shifting on the gating results.

Data set	Test case	Frames range		Synchronized frames		
		Primary view	Secondary view	Maximum expected	DTW	Proposed
I	1	1–40	15–40	25	43	21
	2	35–150	1–140	115	152	104
	3	60–150	1–150	90	164	79
III-C	4	20–33	17–30	13	20	11

The results in Table 3 and Fig. 10 show that using the proposed procedure, which introduces a novel approach based on the partial-matching of XRA frame series, is advantageous for several reasons: (1) The procedure satisfies the requirement that the frame correspondence must be frame-to-frame. This is important because at a moment an artery has two projections on two views. (2) The proposed procedure is more robust when phases are shifted between the series of frames acquired from different views since it can skip false candidates. These two aspects are important since producing false frame pairs during the gating (returning two or more corresponding frames from the secondary view for one matching frame from the primary view and vice-versa) further results with overestimating and delaying the length of CA beat and demotion of CA dynamics. Considering Case 1 and Case 4 (Table 3), the expected frame pairs were 25 and 13, respectively. The proposed partial-matching method shows tendency to slightly underestimate (21 and 11 pairs) while the concurrent method overestimated (43 and 20 pairs), the length of XRA frames sequence. Since in the clinical practice XRA acquisitions are approximately one-two cardiac beats long, cases 2 and 3 remain untypical. Here, they were considered to confirm our assumption that amount of false pairs is proportional to the acquisition phase and length mismatching if one cannot avoid pairing false candidates. On the other side, for the proposed method, false pairs remain low and uncorrelated with the mentioned mismatching between two XRA sequences.

### Vessel centerline reconstruction

**Figure 11:**
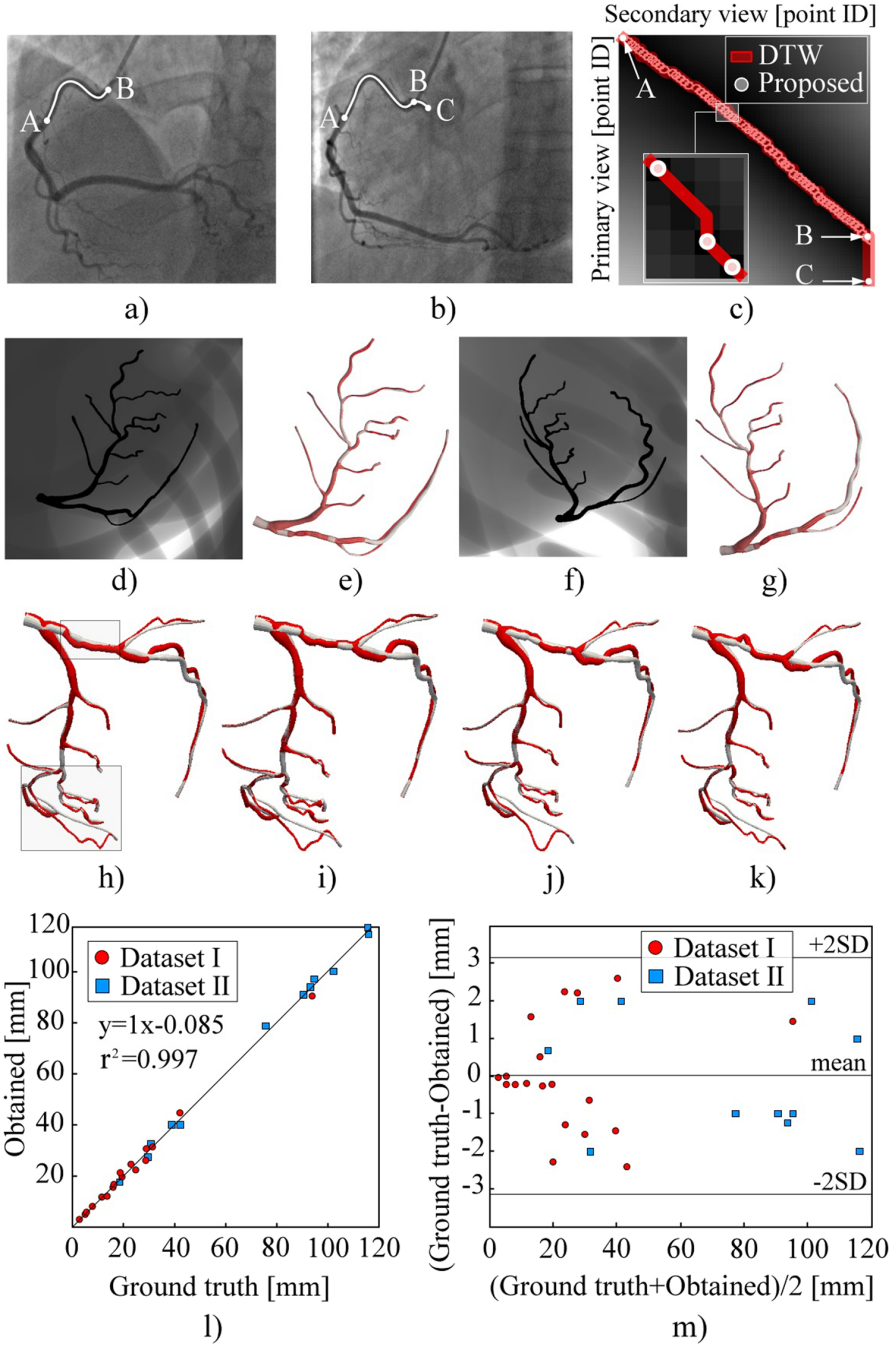
Assessment of CA centerline reconstructions. (**a–k**) Demonstration of the partial-matching ability to skip false candidate points during the segmentation of input centerlines. (**a**) Correctly segmented CA branch. (**b**) Overestimated CA branch (AC was extracted instead of AB). (**c**) Point-correspondence matrix for the XRA views shown on (**a**) and (**b**). (**d–g**) Results from the views of acquisitions. (**h–k**) Results from an arbitrary view during cardiac cycle. (**l**) Correlation of the obtained and true centerlines lengths. (**m**) Bland–Altman plot of the obtained and true branch lengths.

This section assesses the procedure for centerline reconstruction in accuracy and sensitivity to over-under-estimation of CA branch length during the segmentation. An example when a user, or a semi-automated segmentation method, overestimated branch length is given in Fig. 11(a–c) (A–C was marked instead of A–B in Fig. 11(b)).

The proposed method outperforms the conventional methods by skipping the false candidates and only allowing point-to-point matching in Fig. 11(c). Regarding the main branches (determined by two bifurcations, so the problem of overestimating the branch length in 2D should not exist), experiments showed that the proposed method outperformed DTW (or any conventional method unable to skip false points) for both sides and main branches. Two sample projections of the left CA are shown in Fig. 11(d–k) (Video 2 and Video 3). By examining them from the acquisition views in Fig. 11(d–g), only slight differences between the results of the proposed (grey) and DTW (red) methods can be observed. However, when the CA is examined from an arbitrary view (Fig. 11(h–k), Video 4 and Video 5), it is found that only the proposed method resulted in a smooth transition during the complete cardiac cycle.

The assessment of the reconstructed centerlines length is shown in Fig. 11(l,m). The ground-truth lengths of the Dataset I centerlines were known a priori, whereas the centerlines of the Dataset II were computed from the MSCT following the literature^43^. The correlation between the obtained and ground-truth centerline lengths was estimated using Pearson’s correlation coefficient. The obtained lengths in Fig. 11(l) well-correlated with the ground-truth lengths (r^2^ = 0.997); the mean difference, mean absolute difference, and standard deviation of 0.15 mm, 1.283 mm and 1.567 mm, respectively. Paired t-test (*p* = 0.9586 at 0.05 significance level) and Bland-Altman plots (Fig. 11(m)) showed that the errors remained acceptable and did not correlate with the CA length.

### Vascular surface (lumen) reconstruction

**Figure 12:**
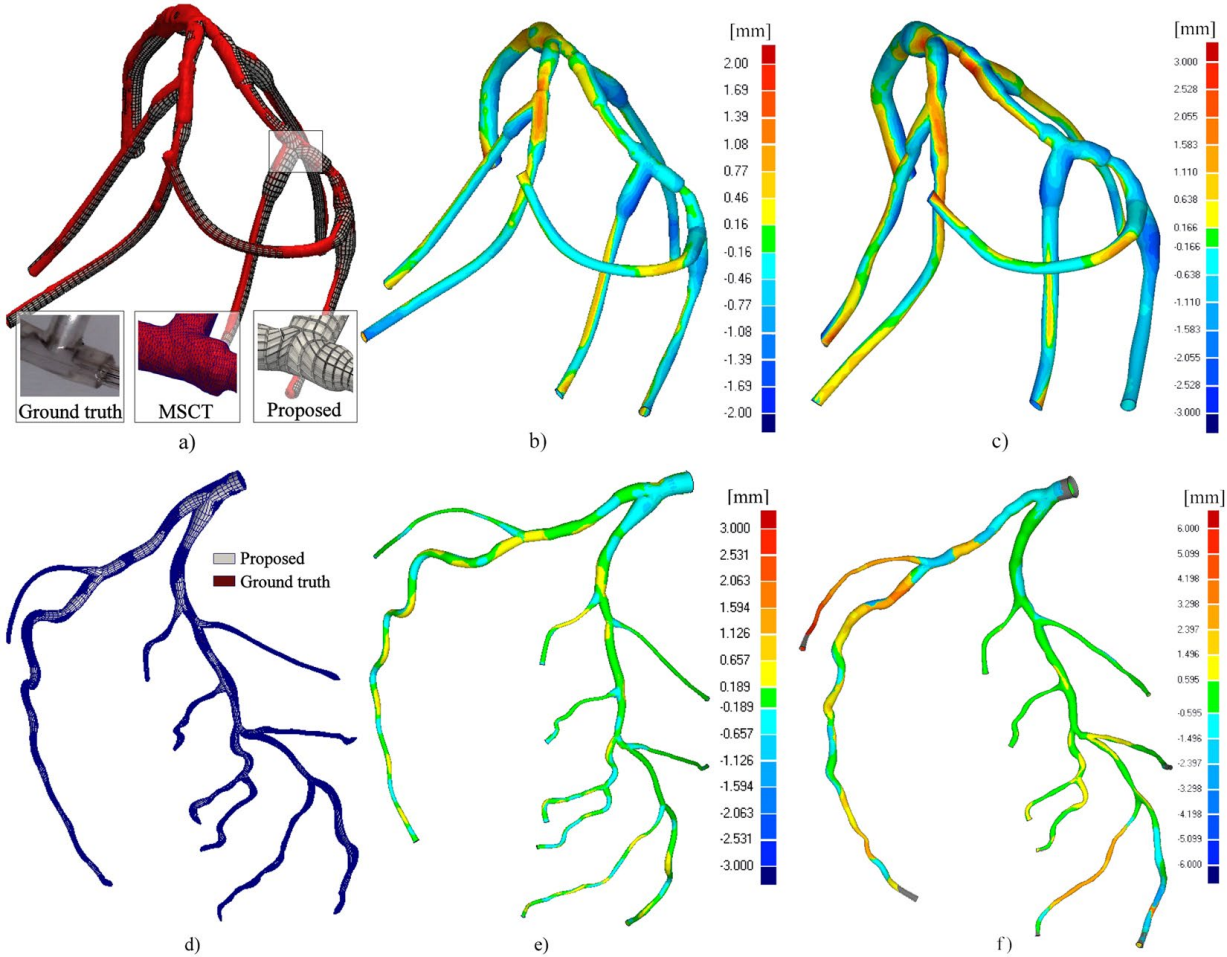
Deviation of the obtained X-ray angiography (XRA) surfaces from the corresponding ground-truth geometry. (**a**–**c**) Physical phantom. (**d**–**f**) Digital XCAT phantom. (**a,d**) Visualization of the mesh quality and level of details. Deviation of the obtained XRA surface from its referent geometry using: (**b,e**) Proposed approach, (**c,f**) Epipolar matching from three views^18^.

The assessment of surface reconstruction was performed by measuring the deviation of the obtained results from the corresponding ground-truth geometry. The referent geometry for Dataset II (obtained by MSCT) is shown with red color in Fig. 12(a,b). The obtained XRA reconstruction (white color) had the maximum surface deviations of 2.85/−2.66 mm at the inlet. The average surface deviations were +0.52 and −0.53 mm, with a standard deviation of 0.68 mm (the maximum Hausdorff distance was 2.35 mm, with the average value of 0.6 mm and the standard deviation of 0.4). A more challenging example is Dataset I shown in Fig. 12(d,e). The maximum surface deviation was +3.47/−1.83 mm, whereas the average surface deviations from the ground-truth data were 0.233/−0.202 mm, with a standard deviation of 0.318 mm (the maximum Hausdorff distance was 2.39 mm, with the average value of 0.52 mm and the standard deviation of 0.38). Although in practice clinicians are usually only interested in reconstructing the main epicardial vessels susceptible to interventional treatment, in this study the complete trees were considered for the reconstruction to evaluate the performance of the method when the view angle is not optimal for each branch. According to Fig. 12(e), good matching was obtained for the left main (LM), proximal circumflex (LCx) and proximal left anterior descending (LAD).

In order to additionally assess our procedure, we performed reconstructions using the epipolar matching for centerline reconstruction while the lumen surface was reconstructed using the multi-view approach^18^. The results obtained for static and dynamics phantom are shown on Fig. 12(c) and Fig. 12(f) (in both cases we used three XRA projections)^18^. For the static phantom, we obtained the maximum surface deviation was +2.86/−3.13 mm, whereas the average surface deviations from the ground-truth data were 0.630/−3.696 mm, with a standard deviation of 0.848 mm. Regarding the MSCT phantom, we obtained the maximum surface deviation, which was +6.026/−3.030 mm, whereas the average surface deviations from the ground-truth data were 1.076/−0.455 mm, with a standard deviation of 1.136 mm. The obtained results showed that the proposed partial matching slightly outperformed the epripolar matching procedure for reconstructing main-short segments of CA tree while significant improvements were obtained for long-curved branches.

From the image based modeling viewpoint, the major flaw of previous approaches was that they deliver the reconstruction results as a triangulated point cloud of fixed density and unguaranteed quality (Table 1). When the aim of reconstruction is the subsequent numerical modeling of CA, the point-cloud meshes require an afterwards manual editing that commonly surpasses the time and effort invested into performing the reconstruction. In the present study we introduce a novel NURBS-based meshing approach that enables us parameterized varying the mesh density over branches (both radial and longitudinal). For the considered data sets, we report that the quality of generated quadrilateral and hexahedral meshes was acceptable, since all generated elements had Jacobian >0.850. Therefore, there was no need for further manual editing of the obtained meshes.

**Figure 13:**
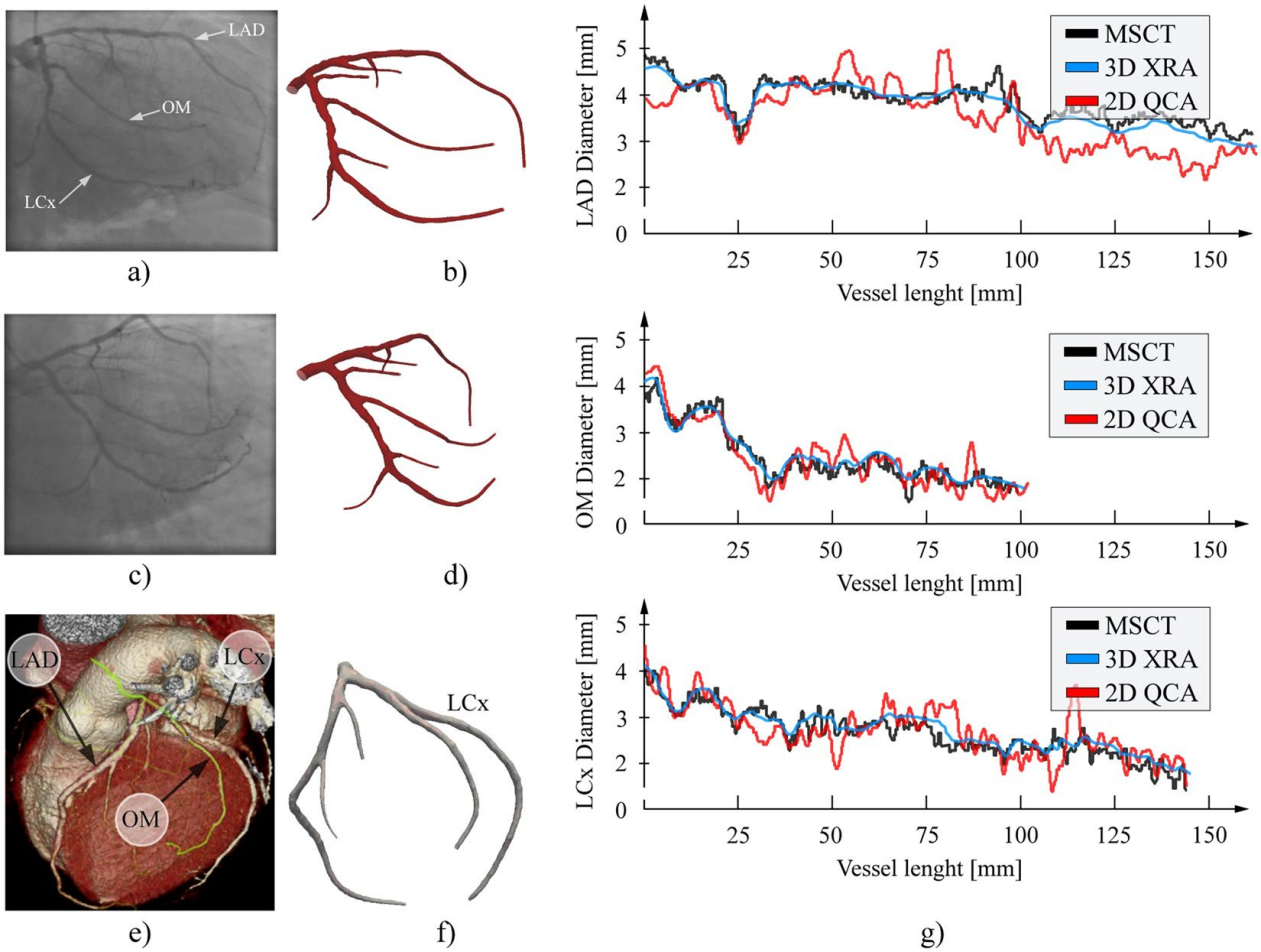
Assessment of the vessel diameters approximation using the clinical data. (**a**–**d**) Two-view XRA (inputs and output surface). (**e**) MSCT scan of the considered CA. (**f**) Surface reconstructed from the MSCT. (**g**) Comparison of the obtained diameters along the main CA branches (LA, LCx and OM).

Beside phantoms, we additionally considered one left CA subjected for both XRA and MSCT with aim to quantitatively compare our results with the geometry obtained from MSCT scans (Fig. 13a–f). Since CAs are highly dynamic vessels that moves and do not deform periodically over time, it is not recommended to compare the overall reconstructed surfaces (due to the inability to capture both acquisitions at the same cardio-respiratory moment). Therefore, we assumed that the vessel cross sections and length are constant over time – so that we can compare the approximated diameters over the branches. The obtained diagrams for the three main branches (LCA, LCx and OM) are given on Fig. 13g. To minimize over shortening, the 2D QCA was performed using the optimal viewpoints for each branch separately. MSCT diameters were computed as mean distance between the cross-section center of gravity and the cross-section contour points (obtained by slicing the triangulated CA surface perpendicularly to the computed branch centerline). Considering Fig. 13g, it may be found that 3D XRA showed good matching with the MSCT approximation of vessel diameters, especially compared to the traditional 2D QCA.

### Sensitivity on various types of lumen constrictions and angulation between XRA views

Finally, the procedure robustness was assessed by varying the angulation between XRA projections and by varying the shape of CA lumen narrowing. Starting from the RAO90 to LAO90 view, 19 projections (Fig. 13(a–c)) of the phantom with stenosis (uniform constriction) and plaque (constriction is not uniform, so that the cross-section has a concave shape) were generated using the XCAT framework. Despite multiple-view XRA is commonly related to the rotational angiography (which was not in the scope of this study), we used the generated XRA views to demonstrate extensibility of our procedure to the polygonal approximation that enables obtaining more realistic representation of CA cross-sections when multiple views are available. Additionally, we aimed to compare these multiple-view results with two-view results in order to quantitatively assess dependency on optimal selection of XRA views.

**Figure 14:**
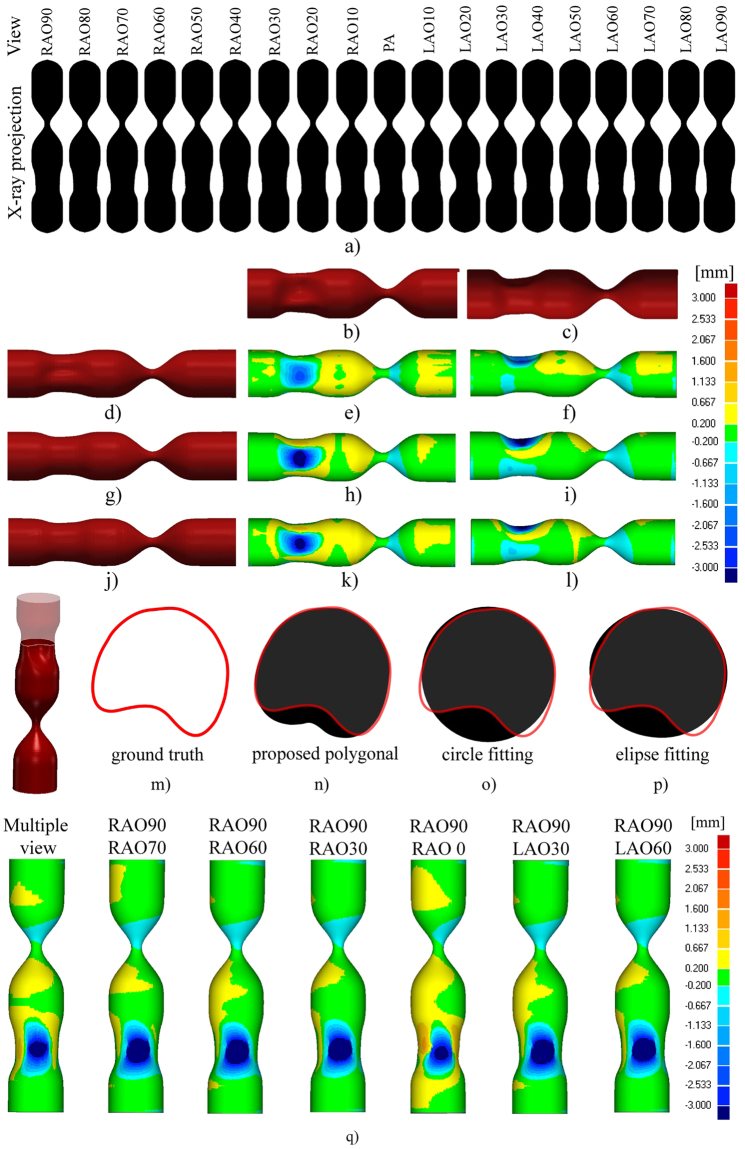
Sensitivity to lumen constrictions and angulation between XRA views. (**a**) Preview of the total 19 XRA views considered for reconstruction, and the numbers above the projections indicate primary angle (PA) right anterior oblique (RAO)/left anterior oblique (LAO). (**b**) Front and (**c**) Top view of the ground-truth surface (phantom). (**d**–**f**) Surface obtained by polygonal approximation vs. ground truth. (**g**–**i**) Surface obtained by circle fitting vs. ground truth. (**j**–**l**) Surface obtained by ellipse fitting vs. ground truth. (**m**–**p**) Comparison of the cross-section assessment approaches for reconstruction of the concave (plaque) region. (**q**) Influence of angle between the X-ray angiography (XRA) views on the accuracy.

The multiple-view reconstructions were obtained by using the polygonal (Fig. 14(d–f)), circles (Fig. 14(g–i)) and ellipses approximation (Fig. 14(j–l)). For the rounded regions, all three approaches provided promising results, with the circle-fitting method exhibiting a slightly higher accuracy. For the plaque region, the polygonal approximation outperformed the other two methods providing the ability to recover the concave (plaque) cross-sections (Fig. 14(m–p)).

Regarding the two-view approach, which is the subject of this study, it may be found that obtained results were comparable to the multi-view approach. The circle-fitting approach showed to be the most robust and least sensitive on variations of the angulation between the views – there was no significant loss of the accuracy with changing the angle between two views (in the range of 20–90 degrees, see Fig. 14q). Furthermore, we found that the ellipse-fitting approach could produce inaccurate results in situations when the angle between the two XRA views is small (less than 30 degrees) because in such situations ellipse fitting could lead to sheared and stretched cross-sections (ellipses) for a given set of points as shown by an example marked with blue color in Fig. 5d. On the basis of these findings, we recommend the circle fitting when only two views are available, using ellipse fitting when at least three views (with spans of at least 30 degrees between them) are available, and using a polygonal approximation when at least six views (with spans of 30 degrees, for example, from RAO90 to LAO90) are available.

## Conclusions and Ongoing work

The present study proposed novel methods for modelling of CA from uncalibrated angiographic X-ray projections. Extensive validations using digital, physical, and clinical datasets showed promising results and potential for clinical validation and application on a wider scale. Despite clinical studies, the proposed procedures are suitable for *in silico* physiology studies, such as virtual calculation of the functional severity of a coronary artery stenosis^44,45^, plaque progression^33^ and studying CA response to stent implantation^46,47^. Our further work will also aim at integrating other imaging techniques, such as intravascular ultrasound^33^ and optical coherence tomography^42,48^. The complex CA trees were reconstructed by decomposing the tree in branches making the proposed framework easy to be parallelized. Finally, our method delivers the final meshes as NURBS suitable for the recently developed isogeometric finite element analysis connecting numerical-modelling techniques with recent developments in computer-aided design^49^.

## Electronic supplementary material

Supplementary Video 1

Supplementary Video 2

Supplementary Video 3

Supplementary Video 4

Supplementary Video 5

Supplementary Video 6

Supporting Information
