# Supplementary material for: Three-dimensional reconstruction and NURBS-based structured meshing of coronary arteries from the conventional X-ray angiography projection images
Source: Sci Rep. 2018 Jan 26;8:1711. doi: 10.1038/s41598-018-19440-9 (PMC5786031; doi:10.1038/s41598-018-19440-9)
Supplement: Supplementary file 7 — Supporting Information [file 41598_2018_19440_MOESM7_ESM.pdf]

Three-dimensional reconstruction and NURBS-based structured meshing of coronary arteries from the conventional X-ray angiography projection images

Arso M. Vukicevic[1,4,5*], Serkan Çimen[2], Nikola Jagic[3], Gordana Jovicic[1], Alejandro F. Frangi[2], Nenad Filipovic[1,5,*]

[1] Faculty of Engineering Sciences, University of Kragujevac, Serbia.

[2] Center for Computational Imaging & Simulation Technologies in Biomedicine, Electronic & Electrical Engineering Department, The University of Sheffield, Sheffield, UK.

[3] Faculty of Medicine, University of Kragujevac.

[4] Research and Development Center for Bioengineering, Kragujevac, Kragujevac, Serbia

[5] Faculty of Information Technology, Belgrade Metropolitan University, Belgrade, Serbia.

[*] Corresponding authors e-mail: arso_kg@yahoo.com, fica@kg.ac.rs.

Appendix S1: Nomenclature

Nomenclature legend

$\mathbf{x}$	–	Data vector-array,	(bold lowercase)
$\mathbf{X}$	–	Matrix,	(bold uppercase)
$\vec{\mathbf{x}}$	–	Point in 2D space (at X-ray plane detector),	(bold lowercase with vector-arrow)
$\vec{\mathbf{X}}$	–	Point in 3D space,	(bold uppercase with vector-arrow)
x	–	Constant variable,	(regular lowercase)
x	–	Scalar variable,	(italic lowercase)
$\mathrm{X}()$	–	Scalar function, result is scalar variable (x),	(regular uppercase)
$\vec{\mathrm{x}}()$	–	Vector function, result is point in 2D ($\vec{\mathbf{x}}$),	(regular lowercase with vector-arrow)
$\vec{\mathrm{X}}()$	–	Vector function, result is point in 3D space ($\vec{\mathbf{X}}$);	(regular uppercase with vector-arrow)

Notes:

- **Bold** indicates data structure; regular style indicates variable or function.
- UPPERCASE indicates that data or computation is in 3D; lowercase indicates that data is in 2D DICOM plane.
- Vector-arrow indicates that type of function or data is point (2D lowercase or 3D UPPERCASE).

At the implementation level these should be different classes (data types).

Nomenclature

n –Number of point samples used for the device calibration

s –Number of vessel centreline samples

$2s$ –Number of vessel borders samples

m –Number of frames acquired from primary view

p –Number of frames acquired from secondary view

$\vec{\mathbf{q}}_{i,j}^{k} \left\{ i = 1,2;\ j = 1,n;\ k = 1,\frac{m\ \ for\ i = 1}{p\ \ for\ i = 2} \right\}$ –Point samples used for calibration

$\vec{\mathrm{c}}_{i,j}^{k} \left\{ i = 1,2;\ j = 1,s;\ k = 1,\frac{m\ \ for\ i = 1}{p\ \ for\ i = 2} \right\}$ –Parameterized vessels centrelines

$\vec{\mathrm{b}}_{i,j}^{k} \left\{ i = 1,2;\ j = 1,2s;\ k = 1,\frac{m\ \ for\ i = 1}{p\ \ for\ i = 2} \right\}$ –Parameterized vessels borders (two for each centreline)

κ –Pixel spacing at 2D X-ray plane detector

α –Primary angle: Right Anterior Oblique (RAO) / Left Anterior Oblique (LAO)

β –Secondary angle: Cranial (CRA) / Caudal (CAU)

d_{SOD} –Distance from patient to X-ray source

d_{SID} –Distance from X-ray source to intensifier plane

$\mathbf{M}$ –C-arm rotation matrix

$\vec{\mathbf{F}}$ –Position of X-ray source in 3D

$\vec{\mathbf{O}}$ –Position of intensifier plane origin in 3D

$\vec{\mathbf{p}}$ –Coordinates of an arbitrary vessel centreline point in the intensifier (DICOM) 2D plane

$\vec{\mathbf{P}}$ –Coordinates of an arbitrary vessel centreline point in the 3D

$\vec{\mathbf{p}}_i\left(\vec{\mathbf{P}}\right)$ –Projection of $\vec{\mathbf{P}}$ back on the intensifier plane for i -th view

$\mathbf{\Delta\theta}$ –Intensifier 3D rotation around its origin $\vec{\mathbf{O}}$

$\mathbf{R}_{\mathbf{\Delta\theta}}$ –Rotation matrix computed from three components in rotation vector $\mathbf{\Delta}\vec{\mathbf{O}} = \left[\Delta o_x \quad \Delta o_y \quad \Delta o_z \quad 1\right]^{\mathrm{T}}$

$\mathbf{\Delta}\vec{\mathbf{O}} = \left[\Delta o_x \quad \Delta o_y \quad \Delta o_z \quad 1\right]^{\mathrm{T}}$ –Intensifier 3D translation from its origin $\vec{\mathbf{O}}$

$\vec{\mathbf{I}}_1$ –Isocenter of primary view

$\vec{\mathbf{I}}_2$ –Isocenter of secondary view

$\mathbf{\Delta i}$ –Translation of the secondary view isocenter $\vec{\mathbf{I}}_2$ from the global origin

$\overset{*}{\mathbf{XA}}\left\{\alpha_2, \beta_2, \mathbf{\Delta\theta}, \mathbf{\Delta}\vec{\mathbf{O}}, \mathbf{\Delta}\vec{\mathbf{i}}\right\}$ –List of device parameters optimized during calibration

F –Cost-function used during the calibration of device parameters $\overset{*}{\mathbf{XA}}$

$\mathbf{D}$ –Cost-matrix for finding correspondence between two series

ξ –Function for calculating values in the cost-matrix

δ –Cost-value for skipping elements from two matching series

$\overset{*}{\varepsilon}_{\min}$ –Calibration error

$\overset{*}{\mathbf{CF}}$ –List of corresponding frames

$\vec{\mathrm{C}}(t)$ –Length-parameterized vessel centreline, $t \in \{0,1\}$

$\vec{\mathbf{C}}(t)$ –Arbitrary 3D point along the parameterized vessel centreline $\vec{\mathrm{C}}(t)$, $t \in \{0,1\}$

$\vec{T}(t)$ –Tangent of the centreline $\vec{\mathrm{C}}(t)$ at point $\vec{\mathbf{C}}(t)$, $t \in \{0,1\}$

$\vec{N}(t)$ –Normal of the centreline $\vec{\mathrm{C}}(t)$ at point $\vec{\mathbf{C}}(t)$, $t \in \{0,1\}$

$\vec{B}(t)$ –Binormal of the centreline $\vec{\mathrm{C}}(t)$ at point $\vec{\mathbf{C}}(t)$, $t \in \{0,1\}$

$\vec{\mathrm{c}}_{\mathrm{i}}(t_i)$ –Parameterized vessel centreline on i -th projection, $t_i \in \{0,1\}$ (note that $t \neq t_i$)

$\vec{\mathbf{c}}_{\mathrm{i}}(t_i)$ –Arbitrary point along the parameterized vessel centreline $\vec{\mathrm{c}}_{\mathrm{i}}(t_i)$ on i -th projection, $t_i \in \{0,1\}$ ($t \neq t_i$)

$\vec{\mathrm{t}}_i(t_i)$ –Tangent of the centreline $\vec{\mathrm{c}}_{\mathrm{i}}(t_i)$ at the point $\vec{\mathbf{c}}_{\mathrm{i}}(t_i)$, $t_i \in \{0,1\}$ ($t \neq t_i$)

$\vec{\mathrm{n}}_i(t_i)$ –Normal of the centreline $\vec{\mathrm{c}}_{\mathrm{i}}(t_i)$ at the point $\vec{\mathbf{c}}_{\mathrm{i}}(t_i)$, $t \in \{0,1\}$ ($t \neq t_i$)

$\vec{\mathbf{b}}_{i,1}, \vec{\mathbf{b}}_{i,2}$ –Intersections of centreline normal $\vec{n}_i(t_i)$ with vessel borders on i -th projection

$\vec{\mathbf{P}}_{a,i}$, $\vec{\mathbf{P}}_{b,i}$ –Points $\vec{\mathbf{b}}_{i,1}, \vec{\mathbf{b}}_{i,2}$ positioned in 3D

$\vec{L}_1(\vec{F}_i, \vec{\mathbf{P}}_{\mathbf{a}_i})$, $\vec{L}_2(\vec{\mathbf{C}}(t), \vec{T}(t))$ – lines (X-rays) – $\vec{L}_1(\vec{F}_i, \vec{\mathbf{P}}_{\mathbf{a}_i})$ is defined by the X-ray source point $\vec{F}_i$ and point $\vec{\mathbf{P}}_{\mathbf{a}_i}$ and the second line $\vec{L}_2(\vec{\mathbf{C}}(t), \vec{T}(t))$ was defined by the point $\vec{\mathbf{C}}(t)$ and direction vector $\vec{T}(t)$. $t \in \{0,1\}$

$\vec{\mathbf{A}}_i(s_u, t)$, $\vec{\mathbf{B}}_i(s_u, t)$ –Points that belong to both rays $L_{1/2}$ and vessel lumen (3D surface) for i -th projection, $t \in \{0,1\}$

$\vec{S}_j(u,v)$ –Parameterized NURBS surface of j -th vessel branch lumen defined by centreline $\vec{P}_j(t)$ and patches obtained along the centreline, $u, v \in \{0,1\}$

$\mathbf{B}$ –Bifurcation list that describes connectivity of considered vessel branches

Appendix S2: Pseudo-code

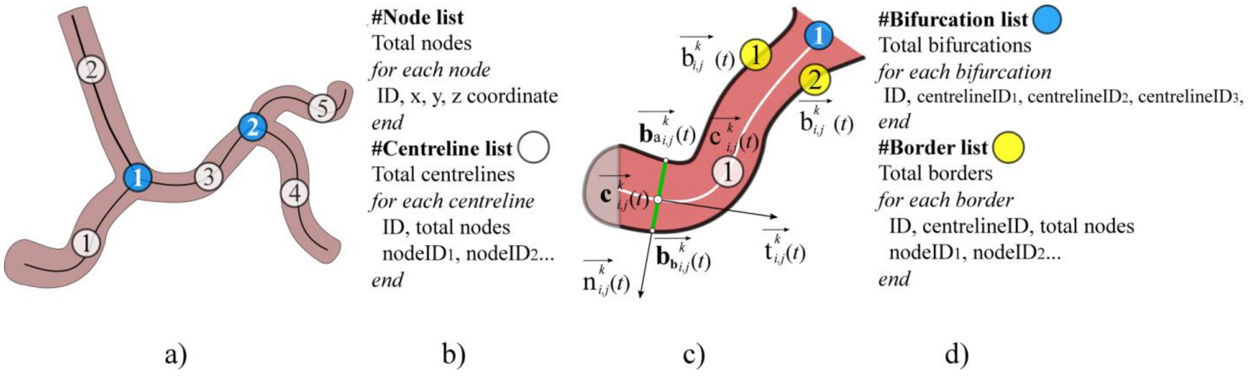

Figure A1. Pre-processing steps and inputs. (a, c) Schematic illustration of the data to be extracted from X-ray angiography (XRA) images; (b, d) Description of input data structures.

The procedure requires two sequences of XRA images acquired from different viewpoints (overall Algorithm 1). After picking the pair of end-diastole frames, a couple (n) of corresponding points $\vec{\mathbf{q}}_{i,j}^{k}\{i=1,2;\ j=1,n;\ k=\text{end-diastole}\}$ were extracted for the purpose of calibration (Algorithm 2) and frames-pairing (Algorithm 3). For the purposes of CA centerlines (Algorithm 4) and surface (Algorithms 5 and 6) reconstruction, the total s vessel centerlines $\vec{c}_{i,j}^{k}\left\{i=1,2;\ j=1,s;\ k=1,\begin{matrix} m \text{ for } i=1 \\ p \text{ for } i=2 \end{matrix}\right\}$ and its corresponding borders $\vec{b}_{i,j}^{k}\left\{i=1,2;\ j=1,2s;\ k=1,\begin{matrix} m \text{ for } i=1 \\ p \text{ for } i=2 \end{matrix}\right\}$ were extracted for each vessel branch; where index i indicates the XRA view, j indicates the point or centerline number, and k indicates the index of the XRA frame in the sequence. Both centerlines $\vec{c}_{i,j}^{k}$ and borders $\vec{b}_{i,j}^{k}$ were defined as parametric curves, so the computation of intersections between the centerline's normal and borders (required for the surface reconstruction) was performed automatically (Fig. A1).

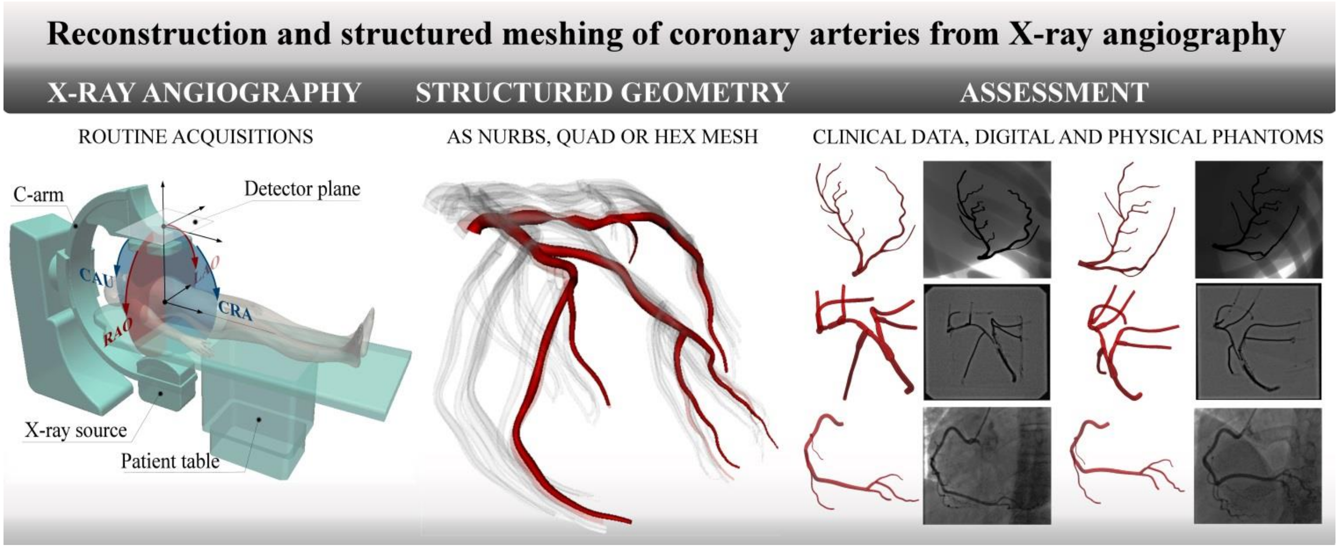

Algorithm 1 Three-dimensional reconstruction of coronary arterial trees from uncalibrated angiographic X-ray projections (overall algorithm)

Input: Total n point samples from two XRA views tracked over m frames from primary view and p frames from secondary view $\vec{\mathbf{q}}_{i,j}^{k}\{i=1,2;\ j=1,n;\ k=\text{end-diastole}\}$.

Input: Total s vessel centrelines $\vec{c}_{i,j}^{k}\left\{i=1,2;\ j=1,s;\ k=1,\frac{m\ for\ i=1}{p\ for\ i=2}\right\}$ tracked over the XRA frames.

Input: Total $2s$ borders (two for each centreline) $\vec{b}_{i,j}^{k}\left\{i=1,2;\ j=1,2s;\ k=1,\frac{m\ for\ i=1}{p\ for\ i=2}\right\}$.

Output: Structured 3D+t mesh of CA tree.

Output: Calibrated XRA device parameters $\overset{*}{\mathbf{XA}}$.

**1**	Find optimal device parameters $\overset{*}{\mathbf{XA}}$ and correspondence $\overset{*}{\mathbf{CP}}$ between the acquired frames (see Algorithm 3)
**2**	**for** k=1 **to** number of corresponding pairs in the list $\overset{*}{\mathbf{CP}}$ **do**
**3**	By using $\overset{*}{\mathbf{XA}}$ and the input data for k-th corresponding pair of frames generate a structured 3D mesh of CA tree (see later Algorithm 6)
**4**	**end for**

Algorithm 2 XRA device calibration using the mathematical model from Section 2.3 and two end-diastole frames (Section 2.4)

**Input:**	Total of n 2D corresponding points from two end-diastole XRA views $\vec{\mathbf{q}}_{i,j}\left\{i=1,2;\ j=1,n\right\}$.
**Input:**	Initial XRA parameters: $\mathbf{XA}\left\{\alpha_2,\beta_2,d_{SOD_{1,2}},d_{SID_{1,2}},\mathbf{\Delta\theta}=[0,0,0],\mathbf{\Delta}\vec{\mathbf{O}}=[0,0,0],\mathbf{\Delta i}=[0,0,0]\right\}$.
**Input:**	Number of GA generations N (in this study $N=100$).
**Input:**	Size of GA population M (in this study $M=30$).
**Output:**	Optimized parameters $\overset{*}{\mathbf{XA}}\{\alpha_2,\beta_2,\mathbf{\Delta\theta},\mathbf{\Delta}\vec{\mathbf{O}},\mathbf{\Delta}\vec{\mathbf{i}}\}$.
**Output:**	Calibration error $\overset{*}{\varepsilon}_{\min}$.

Select an initial population of genomes $g_1^{(l)}, l\in\left\{1...M\right\}$
Set $\varepsilon_{\min}$ to ∞
**for** t=1 to N **do**
**for all** $g_t^{(l)}$ **do**
Set current genome as XRA parameters $g_t^{(l)}\rightarrow\mathbf{XA}$
Reconstruct points $\vec{\mathbf{Q}}_j\{j=1,n\}$ from $\vec{\mathbf{q}}_{i,j}\{i=1,2;\ j=1,n\}$ according to Equations 1–5
Compute error $\varepsilon=\frac{\sum_{i=1}^{2}\sum_{j=1}^{n}\left\lvert\vec{\mathbf{q}}_{i,j}-\vec{\mathbf{q}}_{i,j}\left(\vec{\mathbf{Q}}_j\right)\right\rvert^2}{n}$ according to Equation 6
**if** $\varepsilon<\varepsilon_{\min}$ **then**
$\overset{*}{\varepsilon}_{\min}=\varepsilon_{\min}$, $\overset{*}{\mathbf{XA}}=\mathbf{XA}$
**end if**
**end for**
Reproduce genomes with the lowest training error in the population to form new genomes through crossover and mutation
Replace the worst genomes in the population with the best new genomes
**end for**

Algorithm 3 Partial matching of XRA frame series acquired from two views (Section 2.5)

**Input:**	Total n 2D bifurcation points sampled from two XRA views over m frames from primary view and p frames from secondary view $\vec{\mathbf{q}}_{i,j}^{k}\left\{i=1,2;\ j=1,n;k=1,\frac{m \text{ for } i=1}{p \text{ for } i=2}\right\}$.
**Input:**	Optimized device parameters $\overset{*}{\mathbf{XA}}$. Computed following Algorithm 2.
**Output:**	Optimal correspondence $\overset{*}{\mathbf{CF}}$ between the acquired frames.
**1**	Compute jump-cost C according to Equation 12
**2**	Use referent corresponding end-diastole pair
**3**	Compute jump cost $\xi(\mathbf{a},\mathbf{b})$ according to Equation 8
**4**	Compute cost matrix $\mathbf{D}$ according to Equations 8 and 9
**5**	Find corresponding frames $\overset{*}{\mathbf{CF}} = \mathrm{OSB}(\mathbf{D},\delta)$ according to Equation 7

**Algorithm 4** Reconstruction of vessel centreline from its two corresponding projections (Section 2.6)	
**Input:**	Calibrated device parameters $\overset{*}{\mathbf{XA}}$.
**Input:**	Centreline point samples from two corresponding frames $\vec{\mathbf{c}}_{i,j}\left\{i=1,2;\ j=1,\begin{matrix} m\ \ for\ i=1 \\ p\ \ for\ i=2 \end{matrix}\right\}$, where m and p indicates number of points sampled from primary view and secondary view.
**Output:**	Parameterized vessel centreline in 3D $\vec{\mathrm{C}}(t)$.
**1**	Compute jump cost $\xi(\mathbf{a},\mathbf{b})$ according to Equation 10
**2**	Compute cost matrix $\mathbf{D}$ according to Equations 10 and 11
**3**	Find correspondences between candidate points $\mathbf{a}=\vec{\mathbf{c}}_{\mathrm{i,j}}\{i=1;\ j=1...g\}$ and $\mathbf{b}=\vec{\mathbf{c}}_{\mathrm{i,j}}\{i=2;\ j=1...h\}$ as $\overset{*}{\mathbf{CP}}=\mathrm{OSB}(\mathbf{D},\delta)$ according to Equation 7
**4**	Define parameterized curve $\vec{\mathrm{C}}(t)$ from obtained q corresponding points $\overset{*}{\mathbf{CP}}$

Algorithm 5 Reconstruction of vessel branch 3D surface $\vec{\mathrm{S}}(u,v)$ from its two projections (Section 2.7)

**Input:**	Calibrated device parameters $\overset{*}{\mathbf{XA}}$ obtained following Algorithm 3.
**Input:**	Parameterized vessel centreline $\vec{\mathrm{C}}(t)$ following Algorithm 4.
**Input:**	Parameters $\mathbf{t}=\{t_1,t_2,....\}$ that define positions of patches along the centreline $\vec{\mathrm{P}}(t)$.
**Input:**	Parameters vessel centrelines $\vec{\mathrm{c}}_i\,\{i=1,2\}$ and their corresponding borders $\vec{\mathrm{b}}_{i,j}\,\{i=1,2;\, j=1,2\}$ from two corresponding XRA views.
**Output:**	Parameterized 3D NURBS surface $\vec{\mathrm{S}}(u,v)$ of the vessel.

**1**	**for each** t **in** vector $\mathbf{t}$ **do**
**2**	At point $\vec{\mathbf{C}}(t)$ define a trihedron by the curve's tangent $\vec{\mathrm{T}}(t)$, normal $\vec{\mathrm{N}}(t)$ and binormal $\vec{\mathrm{B}}(t)$
**3**	**for** i = 1 **to** 2 (or more XRA views available) **do**
**4**	Consider centreline point $\vec{\mathbf{C}}(t)$ and its corresponding 2D point $\vec{\mathbf{c}}_i(t_i)$ on the i-th view
**5**	Calculate normal $\vec{\mathrm{n}}_i(t_i)$ of centreline curve at point $\vec{\mathbf{c}}_i(t_i)$
**6**	Calculate points $\vec{\mathbf{p}}_{\mathbf{a}_i}(t_i)$ and $\vec{\mathbf{p}}_{\mathbf{b}_i}(t_i)$ as intersection of normal $\vec{\mathrm{n}}_i(t_i)$ with borders $\vec{\mathrm{b}}_{i,j}$
**7**	Calculate $\vec{\mathbf{P}}_{\mathrm{a,i}}(t)$ from $\vec{\mathbf{p}}_{\mathrm{a}_i}(t_i)$ according to Equation 3
**8**	Define line $\vec{\mathrm{L}}_1(\vec{\mathrm{F}}_i,\vec{\mathbf{P}}_{\mathbf{a}_i})$ using X-ray source point $\vec{\mathbf{F}}_{\mathbf{i}}$ and point $\vec{\mathbf{P}}_{\mathbf{a}_i}(t)$
**9**	Define second line $\vec{\mathrm{L}}_2(\vec{\mathbf{C}}(t),\vec{\mathrm{T}}(t))$ with point $\vec{\mathbf{P}}(\mathbf{t})$ and direction vector $\vec{\mathrm{T}}(t)$
**10**	Find $\vec{\mathbf{A}}_i(s_u,t)$ by solving Equations 1–5for lines L_1 and L_2
**11**	Find $\vec{\mathbf{B}}_i(s_u,t)$ by repeating steps 8–11 for $\vec{\mathbf{p}}_{\mathbf{b}_i}(t_i)\rightarrow\vec{\mathbf{P}}_{\mathbf{b}_i}(t)$
**12**	Project $\vec{\mathbf{A}}_i(s_u,t)$ and $\vec{\mathbf{B}}_i(s_u,t)$ to normal–binormal plane of the trihedron
**13**	**end for**
**14**	Depending on the available projection numbers, fit either a circle, ellipse, or polygonal t-th patch on the points in the normal–binormal plane of the trihedron
**15**	**end for**
**16**	Interpolate each of q patches with w points, and use them to construct NURBS surface $\vec{\mathrm{S}}(u,v)$ according to Equation 18

Algorithm 6 Reconstruction of CA tree mesh from its two corresponding projections (Section 2.9)

**Input:**	Device parameters $\overset{*}{\mathbf{XA}}$ calibrated following Algorithm 3.
**Input:**	Total s vessel centrelines parameterized with B-splines $\vec{\mathrm{C}}_j(t)\{j=1,s\}$ following Algorithm 4.
**Input:**	Bifurcations list describing the connectivity between the CA trees' branches (centrelines). Example: For CA tree composed of tree branches $\vec{\mathrm{C}}_1$ (inlet), $\vec{\mathrm{C}}_2$ (outlet), $\vec{\mathrm{C}}_3$ (outlet), and one bifurcation, the bifurcation list is $\mathbf{B}=\begin{bmatrix}1 & 2 & 3\end{bmatrix}$.
**Output:**	Reconstructed CA tree.

```
for t = 1 to the total number of bifurcations in B do
    Direct trihedrons orientation of the output branches C_{B_t(2)} and C_{B_t(3)} according to the end-trihedron of the input branch C_{B_t(1)} (see Figure 7 c)
end for
for j = 1 to the total number of branches s do
    Reconstruct vessel surface S_j(u,v) from its two corresponding projections following Algorithm 5 (see Figure 7 d)
end for
for t = 1 to the total number of bifurcations in B do
    Trim outlet-side of the input branch S_{B_t(1)} and inlet-side of the output branches S_{B_t(2)} and S_{B_t(3)} following the pattern in Figure 7 e and f
end for
```
